# Genetic Addiction Risk Score (GARS): Molecular Neurogenetic Evidence for Predisposition to Reward Deficiency Syndrome (RDS)

**DOI:** 10.1007/s12035-014-8726-5

**Published:** 2014-05-31

**Authors:** Kenneth Blum, Marlene Oscar-Berman, Zsolt Demetrovics, Debmalya Barh, Mark S. Gold

**Affiliations:** 1 Department of Psychiatry & McKnight Brain Institute, University of Florida College of Medicine, Gainesville, FL USA; 2 Departments of Psychiatry, Neurology, and Anatomy & Neurobiology, Boston University School of Medicine, and Boston VA Healthcare System, Boston, MA 02118 USA; 3 Department of Holistic Medicine, National Institute for Holist Addiction Studies, North Miami Beach, FL USA; 4 Human Integrated Services Unit University of Vermont Center for Clinical & Translational Science, College of Medicine, Burlington, VT USA; 5 Dominion Diagnostics, LLC, North Kingstown, RI USA; 6 Department of Addiction Research & Therapy, Malibu Beach Recovery Center, Malibu Beach, CA USA; 7 Department of Clinical Neurology, PATH Foundation, New York, NY USA; 8 Institute of Integrative Omics and Applied Biotechnology, Nonakuri, Purba Medinipur, West Bengal, India; 9 IGENE, LLC, Austin, TX USA; 10 Department of Clinical Psychology and Addiction, Eötvös Loránd University, Institute of Psychology, Budapest, Hungary

**Keywords:** Genetic Addiction Risk Score (GARS)™, Polymorphisms, brain reward circuitry, Reward Deficiency Syndrome (RDS), Neurogenetics

## Abstract

We have published extensively on the neurogenetics of brain reward systems with reference to the genes related to dopaminergic function in particular. In 1996, we coined “Reward Deficiency Syndrome” (RDS), to portray behaviors found to have gene-based association with hypodopaminergic function. RDS as a useful concept has been embraced in many subsequent studies, to increase our understanding of Substance Use Disorder (SUD), addictions, and other obsessive, compulsive, and impulsive behaviors. Interestingly, albeit others, in one published study, we were able to describe lifetime RDS behaviors in a recovering addict (17 years sober) blindly by assessing resultant Genetic Addiction Risk Score (GARS™) data only. We hypothesize that genetic testing at an early age may be an effective preventive strategy to reduce or eliminate pathological substance and behavioral seeking activity. Here, we consider a select number of genes, their polymorphisms, and associated risks for RDS whereby, utilizing GWAS, there is evidence for convergence to reward candidate genes. The evidence presented serves as a plausible brain-print providing relevant genetic information that will reinforce targeted therapies, to improve recovery and prevent relapse on an individualized basis. The primary driver of RDS is a hypodopaminergic trait (genes) as well as epigenetic states (methylation and deacetylation on chromatin structure). We now have entered a new era in addiction medicine that embraces the neuroscience of addiction and RDS as a pathological condition in brain reward circuitry that calls for appropriate evidence-based therapy and early genetic diagnosis and that requires further intensive investigation.

## The Role of Dopaminergic Genetics in Reward Dependence

**Fig. 1:**
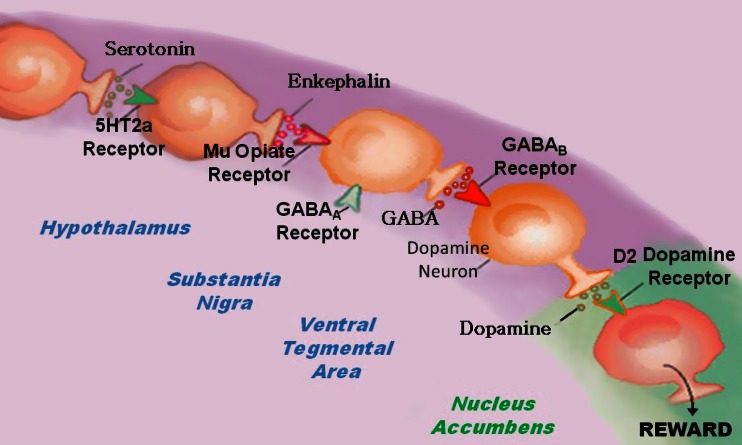
Brain Reward Cascade [14, 15]. In this cascade, stimulation of the serotonergic system in the hypothalamus leads to the stimulation of delta/mu receptors by serotonin to cause a release of enkephalin. Activation of the enkephalinergic system induces an inhibition of GABA transmission at the substania nigra by enkephalin stimulation of mu receptors at GABA neurons. This inhibitory effect allows for the fine-tuning of GABA activity. This provides the normal release of dopamine at the projected area of the NAc [14, 15]

Neurotransmitter interactions regulate brain reward circuitry that result in the release of dopamine (DA) in the major loci for feelings of well-being and reward, the nucleus accumbens (NAc) part of the mesolimbic system of the brain. The inter-relationship of at least four important neurochemical pathways: serotonergic, endorphinergic, GABAergic, and dopaminergic constitute the “brain reward cascade” (see Fig. 1) a natural sequence of events that produce feelings of well being. These activities including: the synthesis, vesicle storage, metabolism, release, and function of neurochemicals [1] are regulated by genes, and their expression, in terms of messenger RNA-directed proteins. Thus, genetic testing is a potential window that can be used to identify the specific neurochemistry of individuals and formulate the best treatment options for them [1–13].

DA is a neurotransmitter with multiple important functions including behavioral effects such as “pleasure” and “stress reduction.” Simply stated, without the normal function of this substance, an individual will suffer from cravings and have an inability to cope with stress. Thus, genetic hypodopaminergic brain function predisposes individuals to seek substances and or behaviors that can be used to overcome this craving state by activating the mesolimbic dopaminergic centers [4, 13]. Psychoactive substances like alcohol, psychostimulants and opiates, and risky behaviors like gambling, overeating and thrill seeking [16] induce the release of neuronal DA into the synapse at the NAc, to overcome the hypodopaminergic state of that individual. Temporary relief from the discomfort and a pseudo sense of well-being is the product of this self-medication [17]. Unfortunately, chronic abuse of psychoactive substances leads to inactivation, or a downregulation, like for example, inhibition of neurotransmitter synthesis, neurotransmitter depletion, formation of toxic pseudo neurotransmitters, and through structural receptor dysfunction. Therefore, substance-seeking and pathological behaviors are both used as a means of providing a feel-good response (a “fix”) to lessen uncontrollable cravings. Individuals who possess reward gene polymorphisms or variations, will, given environmental insult be at risk for impulsive, compulsive, and addictive behaviors. Reward Deficiency Syndrome (RDS) is a term used to embrace and characterize these genetically induced behaviors. Any and all of these pathological behaviors, as well as psychoactive drug-abuse, are candidates for addiction including tolerance and dependence. The behavior or drug of choice by the individual is a function of both genes and environmental factors like availability and peer pressure.

## Brain Reward Cascade Explanation

DA is crucial to the maintenance of natural rewards while the release of DA into NAc synaptic sites is a somewhat complex cascade of reactive activity that involves neurotransmitters and structures in the limbic system [1]. Blum and Kozlowski first proposed the concept of a “brain reward cascade” in 1990 as a cascade of interactive events and mesolimbic function that produces the net release of DA [1] (see Fig. 1). Simply, the interaction of activities in the separate subsystems of the brain’s reward circuitry combine into the much larger global system, and reveal the cascade of neurotransmission, which merges simultaneously and in a specific sequence. When these systems work normally, they result in a feeling of pleasure, well-being, and peace; an imbalance, or deficiency, on the other hand, will cause the system to function abnormally, displacing the sense of well-being with negative feelings like anxiety, anger, and low self-esteem. The need to mask these negative feelings leads to the use of substances such as alcohol and narcotics, meaning that excessive desires are spurred by the need for DA.

The DA pathway arises in the ventral tegmental area (VTA) and culminates in the DA D2 receptors on neurons located in the cell membranes of in the hippocampus and the NAc. Blum and Kozlowski [1] describe a process that begins in the hypothalamus, where the excitatory activities of 5-HT-releasing-neurons cause the release of met-enkephalin, an opioid peptide. The opioid peptide regulates the activity of neurons responsible for the release of gamma-aminobutyric acid (GABA) the inhibitory neurotransmitter at the substantia nigra. When DA-containing neurons in the VTA, and in certain parts of the hippocampus via the amygdala, are disinhibited, DA is allowed to be released into the NAc, permitting the completion of the cascade. If the cascade is functioning correctly, the reward sensation, or the feeling of well-being, is experienced, provided certain basic genetic conditions are fulfilled (see Fig. 1) [1]. However, when the genes that govern the function of the brain reward cascade have polymorphic variations, these risk alleles provide the basis for therapeutic targets.

## RDS and Genetic Antecedents

The development of a blueprint for identifying certain candidate genes and polymorphisms that could negatively impact DA release is based on this understanding of the brain reward cascade [2]. Many genes are involved, and it has been adequately established in association studies and animal research literature that, for example, polymorphisms of the serotonergic-2 A receptor (*5-HTT2a*), DA D2 receptor (*DRD2*), and the catechol-*O*-methyl-transferase (*COMT*) genes (see Genetic Addiction Risk Score (GARS) test), predispose individuals to aberrant RDS behaviors. These behaviors include craving not only for drugs and alcohol but eating and other addictive behaviors such as pathological gambling (see Table 1) [3]. Gene polymorphisms of both 5-HT and DA can result in significantly lower than normal receptor densities. A COMT gene polymorphism can result in increased catabolism of synaptic DA and subsequent reduction of DA function. These are three examples of how the identification of polymorphisms on these three genes can provide a window into an impaired brain reward cascade, and the identification of individuals at high risk can be accomplished. Based on a published [11] mathematical Bayesian approach, it was found that individuals who carry the *Taq* A1 polymorphisms of the *DRD2* have a 74.4 % chance of developing RDS behaviors, given an environmental insult and epigenetic effects (see Table 1).

**Table 1:** Reward deficiency syndrome behaviors (linked with DSM 5)

Addictive behaviors		Impulsive behaviors		Obsessive compulsive behaviors	Personality disorders
Substance related	Non substance related	Spectrum disorders	Disruptive impulsive		
Alcohol	Thrill seeking (novelty)	Attention-deficit hyperactivity	Anti-social	Body dysmorphic	Paranoid
Cannabis	Sexual sadism	Tourette and tic Syndrome	Conduct	Hoarding	Schizoid
Opioids	Sexual masochism	Autism	Intermittent explosive	Trichotillomania (hair pulling)	Borderline
Sedatives/hypnotics	Hypersexual		Oppositional defiant	Excoriation (skin picking)	Schizotypal
Stimulants	Gambling		Exhibitionistic	Non-suicidal self-injury	Histrionic
Tobacco	Internet gaming				Narcissistic
Glucose					Avoidant
Food					Dependant

## Genomics: Evidence-Based Studies

**Fig. 2:**
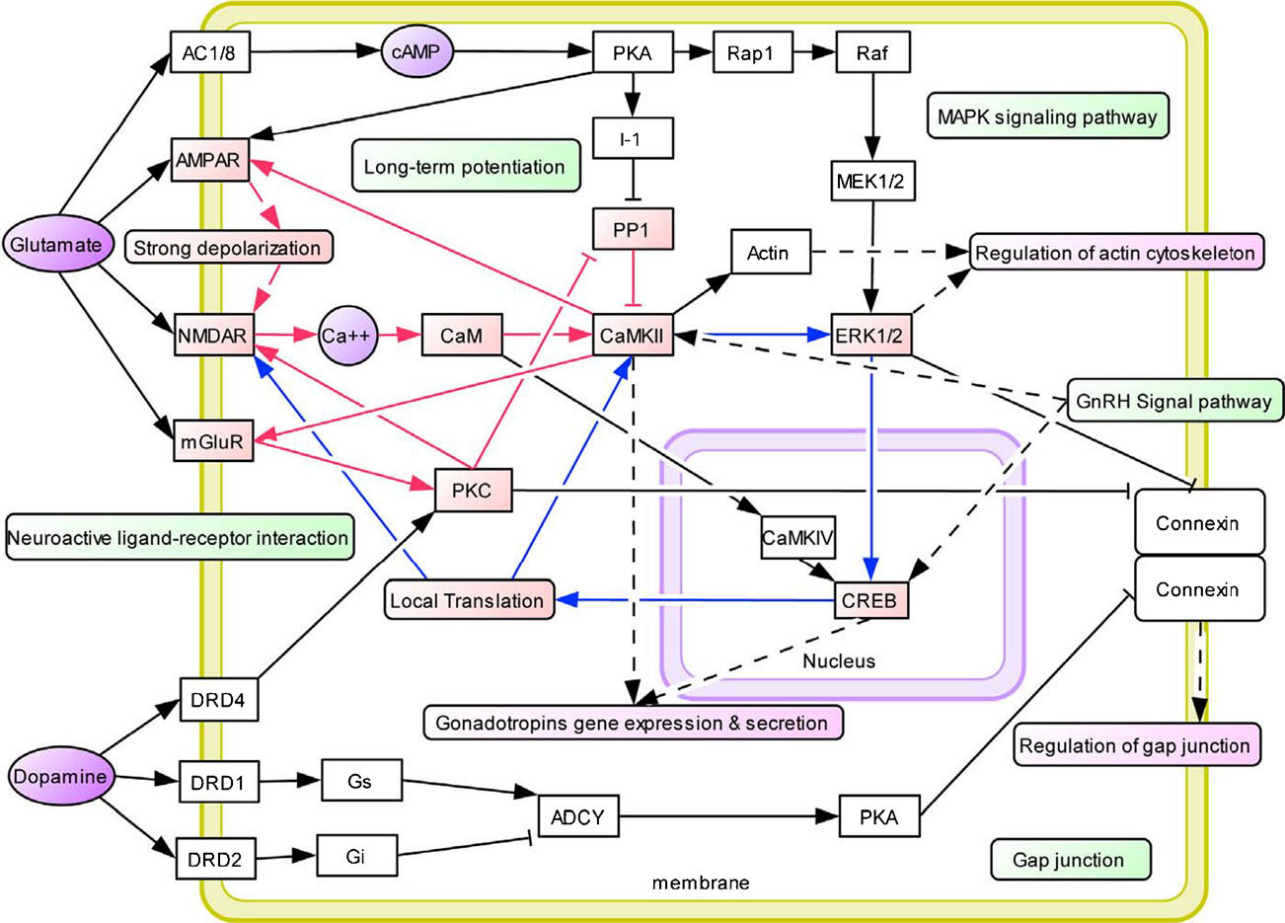
KARG an addiction network map [6]

In general, inconsistencies in the literature involving association studies using single gene analysis prompted Conner [4] and others to evaluate a number of dopaminergic gene polymorphisms as predictors of drug use in adolescents. We cannot ignore the importance of neurochemical mechanisms involved in drug-induced relapse behavior, as suggested by Bossert et al. [5] for understanding the interaction of multiple genes and environmental elements. Using a drug relapse model previously shown to induce relapse by re-exposing rats to heroin-associated contexts, these investigators found that after extinction of drug-reinforced responding in different contexts, re-exposure reinstated heroin seeking. This effect was diminished by inhibition of GABA transmission in the VTA and medial accumbens shell and components of the mesolimbic DA system; this process enhances net DA release into the NAc. Indeed, this fits well with Li’s Knowledgebase for Addiction-Related Genes (KARG) addiction network map [6] (see Fig. 2). Li et al. [6] also stressed the view that drug addiction is a serious problem worldwide with strong genetic and environmental influences. And that a variety of technologies were used to discover genes and pathways that underlie addiction however, each individual technology can be biased and incomplete. Li and his colleagues integrated evidence from peer-reviewed publications 2,343 items between 1976 and 2006 that inked genes and chromosomal regions to addiction by single-gene strategies, microarray, proteomics, or genetic studies. They identified 1,500 human addiction-related genes and developed the first molecular KARG (http://karg.cbi.pku.edu.cn), with a friendly web interface and extensive annotations. Their meta-analysis of 396 genes, each supported by two or more independent items of evidence, leads to the identification of 18 molecular pathways that were statistically significant and covered both upstream signaling events and downstream effects. For four different types of addictive drugs and five significant molecular pathways including two new ones; GnRH signaling pathway and gap junction were identified as common pathways that may underlie shared rewarding and addictive actions. In a hypothetical common molecular network for addiction, they connected the common pathways which linked all of these genes, to both the glutaminergic and dopaminergic pathways.

Following the initial finding of Blum et al. in 1990 [7] showing a positive association of the single gene DRD2 polymorphism in chromosome 11 and severe alcoholism, replication, although favorable has, to date, been fraught with inconsistent results. This has also been true for other complex behaviors [9] when gene-gene and gene-environment interactions are tested the idea that complex gene-relationships may account for inconsistent findings across many different single gene studies is supported [8]. The reasons for inconsistencies in trying to predict drug use are many and varied: they include single gene analysis, poorly screened controls, stratification of population, personality traits, co-morbidity of psychiatric disorders, gender-base differences, positive and negative life events, and neurocognitive dysfunctioning and epigenetic effects [6, 7].

In order to gain a more complex but stronger predictive set of genetic antecedents rather than continue to evaluate single gene associations, we embarked on a study to evaluate multiple candidate genes, especially those linked to the Brain Reward Cascade to predict future drug abuse [1] and identify risk for hypodopaminergic functioning. Although exploratory, the goal is to develop an informative panel based on numerous known risk alleles to provide treatment facilities a means of stratifying patients entering treatment as having a high, moderate, or low, genetic risk prediction.

As noted above, an association between dopaminergic gene polymorphisms and addictive, compulsive, and impulsive behaviors classified as RDS has been revealed in numerous studies. We evaluated subjects derived from two families for a potential association with polymorphisms of the DA D2 receptor gene (*DRD2*), DA D1 receptor gene (*DRD1*), DA transporter gene (*DAT1*), and DA beta-hydroxylase gene (*DBH*). This association if found would demonstrate the relevance of a generalized RDS behavior set, as the phenotype.

An experimental group derived from up to five generations of two independent multiple-affected families *n* = 55 were genotyped and compared to very rigorously screened controls. In addition to these subjects, data related to RDS behaviors was collected from 13 deceased family members. The genotyped family members carried the DRD2 *Taq*1 allele at 78 % the DAT1 10/10 allele at 58 %, the DBHB1 allele at 66 %, and the DRD1 A1/A1 or the A2/A2 genotypes at 35 %. Interestingly, all probands (*n* = 32) from Family A genotyped for the DRD2 gene carried the *Taq*A1 allele (100 %). The experimental positive rate for the DRD2 *Taq*1 allele with an odds ratio of 103.9 (12.8, 843.2) was significantly greater (*X* ^2^ = 43.6, *P* < 0.001). The experimental positive rate for the DAT1 10/10 allele with an odds ratio of 2.3 (1.2, 4.6) was also significantly greater (*X* ^2^ = 6.0, *P* < 0.015). Between the experimental and control positive rates of the DBH, DRD1 A1/A1, or A2/A2 genotypes no significant differences were observed [18].

Patients genetic risk for drug-seeking behavior needs to be evaluated prior to, or upon entry to chemical dependency programs. The importance of genotyping to establish genetic severity for patients undergoing treatment and in danger of relapse is described below in the, as yet, unpublished results from a study of the data from Comprehensive Analysis of Reported Drugs (CARD). We have followed up by evaluating a panel of genes and associated polymorphisms termed GARS in patients with RDS behaviors attending two treatment centers. In this and other studies, we use the GARS for purposes of study identification and commercial testing [9–11].

To determine risk severity of 72 addicted patients, the percentage of prevalence of selected risk alleles was calculated to provide a severity score. We genotyped the patients using a nine reward genes and their polymorphisms (*F* = 18 alleles; *M* = 17 alleles). This panel included: *DRD 2*, *3*, *4*; *MOA-A*; *COMT*; *DAT1*; *5HTTLLR*; *OPRM1*; and *GABRA3* genes. The three severity ratings were: Low severity = 1–36 %; moderate severity = 37–50 %, and high severity = 51–100 %. We studied two distinct treatment populations: Group 1 consisted of 37 addicts from a holistic addiction treatment center in North Miami Beach, Florida, and Group 2 consisted of 35 addicts from Malibu Beach Recovery Center [12]. We are in the process of analyzing 393 subjects using a multi-centered approach across the USA.

However, in the following unpublished experiment, we found risk stratification of the 72 genotyped patients to be as follows: 27 % low risk; 74 % moderate risk, and 4 % severe risk. We are exploring potential risk correlation with the Addiction Severity Index (ASI). Preliminary statistical analysis reveals that with *N* = 277, we found a significant trend whereby allelic risk above the means score associated with the ASI Alcohol Risk Severity Score at *P* < 0.07 (one sided P). Unlike CARD, the GARS could provide potential correlations to ASI to at least the alcohol risk composite score. If this finding is upheld through larger populations, it unequivocally demonstrates that objective genetic polymorphisms could predict clinical outcomes.

We are cognizant that, as the next steps in identifying candidate gene polymorphic associations with RDS as the overall phenotype, we must carefully dissect epigenetic effects such as miRNA and subsequent methylation and/or deacetylation of attached chromatin markers leading to altered gene expression in spite of DNA polymorphisms.

## CARD™ Provides a Rationale for Genetic Testing

The primary reason to include some brief information regarding CARD is to provide a clear indication that genetic testing may provide valuable information about the risk that drives relapse. In an unpublished study but submitted article from our laboratory, a statistical analysis of unidentifiable data from a computer-based program called CARD was used to evaluate treatment adherence in a large clinical cohort from across a number of eastern states in America. This study consisted of 5,703 patients and 11,403 specimens, in various treatment settings across six eastern states. We found significant levels of both non-compliance and lack of abstinence (risk for relapse) during treatment. The CARD engine addresses issues of metabolism as well as contaminants in the production processes of some formulations. It addresses multi-faceted scenarios within and across drug classes, often involving the state of multiple analytes in order to reach a conclusion for each self-reported or prescribed drug. It evaluates thousands of rule sets to determine if the statement associated with each rule set is applicable to the specimen test results and reported drugs that are analyzed.

We are proposing a paradigm shift on the basis of these studies, whereby the predisposition to a risk for RDS (the true phenotype) can be accurately determined by utilizing GARS, and treatment outcome can be assessed by utilizing CARD. These results confirm the putative role of dopaminergic polymorphisms in RDS behaviors.

## The Addiction Phenotype and the Need for Super Controls

The family-based study [18] demonstrates the importance of a nonspecific RDS phenotype and informed an understanding of how evaluating single subset of RDS behaviors, like for example, Tourette’s may lead to spurious results. Rather, the adoption of a nonspecific reward phenotype may be useful in future association and linkage studies involving neurotransmitter gene candidates as utilized in GARS. The putative role of dopaminergic polymorphisms in RDS behaviors is supported by the results [18], although linkage analysis is necessary and the sample size was limited. We believe that using a nonspecific reward phenotype in future association and linkage studies that involve dopaminergic polymorphisms and other neurotransmitter gene candidates may be a necessary paradigm shift.

**Fig. 3:**
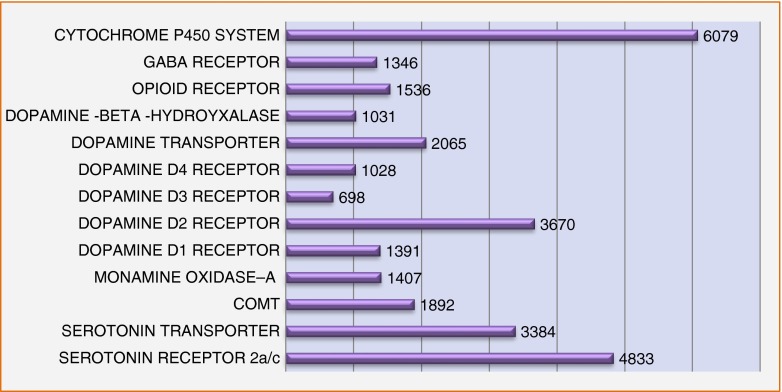
This is a list of Pub Med articles that associate polymorphisms of reward genes with risk of RDS behaviors. For each gene, there are many polymorphisms, and there are multiple receptors for each listed transmitter. The DRD2 gene is the most widely studied as a single receptor type. Reward Gene Publications 3/16/2014

This underscores the problem concerning appropriate controls. While thousands of studies have associated the various reward gene risk polymorphisms for all types of addictive behaviors (including drugs, smoking, alcohol, gambling, sex, shopping) against putative controls, there remains a real need to develop super controls whereby the true phenotype is not just drug addiction per se but the absence of any RDS behavior. To suggest that researchers can provide accurate data by enlisting comparison individuals who are from an unscreened general population as controls, is fraught with an inappropriate and potentially inaccurate assessment. In one, example, assessing the DRD2 A1 allele, we found that while screened controls (eliminating drug and alcohol abuse) in over 3,000 subjects showed a prevalence of approximately 26 %; when we eliminated all RDS behaviors in the probands and family surprisingly we found the DRD2 A1 allele prevalence to be only 3 % [19]. In the current GARS test being cognizant of this issue, we utilize the recognized method of counting risk alleles to provide addiction risk. Below, we provide a chart showing the remarkable Pub Med (3-16-14) list of articles published on each independent gene involving risk polymorphisms in RDS behavior and controls (Fig. 3).

## Theoretical Implications: Substance Abuse and Pain Medications

Understanding that there is a thread between opioid prescribed compounds for pain and addiction liability especially in subjects genetically predisposed to RDS risk provides the rationale to address this growing epidemic globally. The GARS test modified for pain clinics provides an analysis of about 14 genes and associated risk alleles. Thousands of studies in peer reviewed scientific journals have revealed significant associations between certain reward genes, with reward circuitry imbalances in the brain and risk for high substance seeking behavior. The predictive value for just one gene such as the DA D2 receptor gene is as high as 74.4 % as described by Blum et al. [12]. Simply, the occurrence or absence of these single nucleotide polymorphisms may determine a patient’s predisposition to potential treatment outcome and relapse. In addition to addiction risk, it may help guide the physician in determining the use of chronic opioid therapy, and a rationale for continuing urine monitoring consistent with the American College of Occupational and Environmental Medicine (ACOEM) guidelines. Most importantly, ACOEM suggests that genetics are an important factor in pain management, and according to the National Institute on Drug Abuse (NIDA), as well as the American Society of Addiction Medicine (ASAM), genes are responsible for a 60 % contribution toward addictive behaviors. Not surprisingly, according to the American Pain Society, a physician’s ability to predict an opioid abuser is no better than chance (50 %). In fact, Bornstein’s group [20] found that when clinicians’ only urine test patients suspected of medication misuse, they are missing a significant group; up to 72 % and are quick to make wrong judgments. The ACOEM guidelines on Chronic Opioid Therapy suggest “screening for risk of addiction should be performed before starting a long-term opioid treatment in patients with chronic pain”, thus providing the physician with clues about the necessity for increased attention in susceptible patients. If opioid treatment results in pain control, better functioning, and improved health-related quality of life, the treatment should be continued, even in patients susceptible for addiction. However, these patients will need special attention with a focus on compliance, abstinence from other drugs of abuse and with discussion of the potential consequences of chronic treatment of pain with opioids.

Although the principal pain pathways ascend to the brain from the dorsal horn of the spinal cord the control of sensitivity to pain may reside in the mesolimbic system of the brain at the reward center, where gene polymorphisms may impact pain tolerance and/or sensitivity. These polymorphisms may associate with a predisposition to pain intolerance or tolerance to pain. It is hypothesized that the identification of certain gene polymorphisms may provide a unique therapeutic target to assist in pain treatment. Thus, testing for certain candidate genes like the mu receptors and PENK could assist in the design of pharmacogenomic solutions personalized to each patient and guided by their unique genetic makeup [10], with potential for improvement in clinical outcomes [11].

Understanding the role of neurogenetics in pain relief, including pharmacogenomics and nutrigenomic aspects, will pave the way to better treatment for the millions suffering from both acute and chronic pain. We now know that dopaminergic tone is involved in pain sensitivity mechanisms and even buprenorphine outcome response. The identification of certain gene polymorphisms as unique, therapeutic targets may assist in the treatment of pain. Pharmacogenetic testing for certain candidate genes, like mu receptors and PENK, is proposed, as a means to improve clinical outcomes by the provision of treatment. The use of GARS, as described above, to identify clients with high addiction risk by providing valuable information about genetic predisposition to opioid addiction, could become an important frontline approach, on admission to pain clinics.

One notable study evaluated the role of both mu-opioid receptors (MORs) and delta-opioid receptors (DORs) two genes expressed in the VTA that may be involved in the addictive properties of opiates. Researchers David et al. [21] found that intra-VTA morphine self-administration was abolished in knockout MOR gene mice at all doses tested. While male and female WT and DOR−/− mice exhibited self-administration similarly, however, this behavior was disrupted without triggering physical signs of withdrawal by the administration of Naloxone (4 mg/kg) to WT and DOR mutants. An increase in *fos* was associated with Morphine ICSA within the NAc, striatum, limbic cortices, amygdala, hippocampus, lateral mammillary nucleus, and the ventral posteromedial thalamus where high levels of *fos* were expressed exclusively in self-administering WT and DOR−/− mice. Abolition of morphine reward in MOR−/− mice was associated with a decrease in *fos* positive neurons in the mesocorticolimbic DA system, amygdala, hippocampus (CA1), lateral mammillary nucleus, and a complete absence within the ventral posteromedial thalamus. David et al. [21] concluded that (a) ventral posteromedial thalamus MORs, but not DORs, are critical for morphine reward and (b) the role of VTA-thalamic projections in opiate reward warrants further exploration.

Moreover, clinical and laboratory studies have indicated that the MOR gene contributes to inheritable vulnerability to the development of opiate addiction. Polymorphisms that occur naturally have been identified in the MOR gene. Substitutions occur at high allelic frequencies (10.5 and 6.6 %) in two coding regions single nucleotide polymorphisms (SNPs), the A118G and C17T respectively, of the MOR gene. These SNPs cause amino acid changes in the receptor that impact on an individual’s response to opioids and can influence increases or decreases in vulnerability to opiate addiction [22]. Thus, in response to beta-endorphin in cellular assays, the A118G substitution encodes a variant receptor with differences in binding and signal transduction [22]. Finally, to firmly establish the role of MOR in reward and response to buprenorphine, Ide et al. [23] assessed buprenorphine anti-nociception by hot-plate and tail-flick tests, and found that it was significantly reduced in heterozygous mu-opioid receptor knockout (MOR-KO) mice and abolished in homozygous MOR-KO mice. Buprenorphine, on the other hand, was able to establish a conditioned place preference in homozygous MOR-KO, although as the number of copies of wild-type mu-opioid receptor genes was reduced, the magnitude of place preference was reduced. This study revealed that mu-opioid receptors mediate most of analgesic properties of buprenorphine [23]. We are proposing that to determine patient addiction liability, genetic testing should be incorporated into the beginning of Occupational Medical Clinic programs to reduce iatrogenic opioid prescription addiction, and should include both opioid and dopaminergic risk alleles.

## Explanation of Single Nucleotide Polymorphisms

RDS-associated SNPs can be identified by any suitable method, including DNA sequencing of patients diagnosed with one or more RDS behaviors. After validation, newly identified RDS-associated SNPs can be used in the test. As will be appreciated, once identified and validated, the presence, if any, of one or more RDS-associated SNPs in the nucleic acids derived from a biological sample taken from a patient can be determined using any suitable now known or later-developed assay, including those that rely on site-specific hybridization, restriction enzyme analysis, or DNA sequencing. Table 2 lists a number of particularly preferred RDS-associated SNPs, whereby, the detection of which can be used for the GARS test.

**Table 2:** RDS-associated SNPs

Gene	Risk allele	Comment
Dopamine D1 (DRD)	48A	G normal
Dopamine D2 (DRD2)	A1	A2 normal
Dopamine D3 (DRD3)	C	T normal
Dopamine D4 (DRD4)	7R	4R normal
Dopamine Transporter (DAT1)	9R = Fast uptake 10R = slow uptake	Fast DAT could result in hypodopaminergic and slow could result in hyper dopaminergic
Serotonin Transporter (5HTTLLR)	S	Count S not L
Catechol-O-methyl-transferase (COMT)	G	The G allele = Val substation that cause the enzyme COMT which breaks down Dopamine in the synapse too fast. This could also lead to hypodopaminergic trait. The A = Met = normal
Mu opiate receptor (OPRM1)	G	The G allele whereby G = ASP this contributes to addiction to opiates and alcohol. A = ASN normal. Another name is MOR-Mu opiate receptor
GABA A receptor subunit (GABRA3)	181	This 181 snp reduces the sensitivity of the GABA receptor and as such increases the chance for alcoholism and other drugs of abuse. It increases risk for stress induction, which can also cause relapse
MAOA uVNTR	4R = Fast uptake 3R = slow uptake	This is the strange gene. It sits on the mitochondria in the neuron. MAO is involved in the breakdown of dopamine and serotonin. The 4R increases the breakdown and 3R slows the breakdown. Since the gene sits on the X chromosome not the Y chromosome females are XX and males are XY. This means that females have two alleles to count and males only have one
Serotonin 5HTA2 Receptor	C	Alcohol dependent (AD) patients homozygous for C allele had significantly lower age at onset of alcohol problems than subjects having at least one T allele. The results suggest a potential role of the T102C HTR2A polymorphism in development of alcohol dependence
Serotonin 5HTA2 Receptor	1438A allele	Another polymorphism the 5-HT (2A) -1438A allele was significantly more common in patients than controls [0.55 and 0.45, respectively; corrected *P* = 0.042, OR = 1.51 (95 % CI = 1.13–2.03)]

## Serotonin (5-Hydroxytriptamine5-HT) Genes (2AReceptor1438G/a)

Serotonin, also known as 5-hydroxytryptamine or 5-HT, is a neurotransmitter and peripheral NH2 signal mediator that was discovered in the late 1940s. By the early 1950s, neurotransmitter function was identified in the central nervous system of animals. In the late 1950s, there was evidence for 5-HT receptor peripheral heterogeneity, and by 1979, 5-HT binding sites were identified in the brain: 5-HT1 and 5-HT2. 5-HT 2A receptor (5-HT2A) is one of several proteins to which 5-HT binds when brain cells communicate. 5-HT receptors located on the membranes of nerve and other cell types mediate the effects of 5-HT as the endogenous ligand. 5-HT receptors are heptahelical; G protein coupled seven trans-membrane receptors, activated by an intracellular second messenger cascade except for the 5-HT3 receptor, a ligand gated ion channel. The 5-HT receptor contains 471 amino acids in rats, mice, and humans and is widely distributed in peripheral and central tissues. 5-HT receptors mediate contractile responses in a series of vascular smooth muscle preparations. In addition, platelet aggregation and increased capillary permeability following exposure to 5-HT have been linked to 5-HT receptor-mediated functions. Centrally, these receptors are located principally on cells in the cerebral cortex, claustrum, and basal ganglia. 5-HT receptors reduce cyclic adenosine monophosphate (cAMP) and modify the activity and release of other neurotransmitters like glutamate, enkephalin, DA, and GABA. 5-HT2A receptors increase glutamate activity of in many areas of the brain, some of the other 5-HT receptors have the effect of suppressing glutamate. The therapeutic actions that result from increased stimulation of 5-HT receptors in anti-depressant and anxiolytic treatments seems to be opposed by increased stimulation of the 5-HT2A receptors.

## Serotonin Receptors (2A) Genetics

Everyone inherits two copies of the 5-HT2A receptor gene, one from each parent. Small differences in the chemical sequence results, in some people having an adenine (A), switched at the same point for a guanine (G). So a subject can have gene types AA, AG, or GG. According to the US Department of Health and Human Services National Institutes of Health, “whether depressed patients will respond to an antidepressant depends in part, on which version of a gene they inherit.” The chance of a positive response to an antidepressant increase by up to 18 % in those who have two copies of one version of a gene that codes for a component of brain mood-regulation. It is well known that polymorphisms at the 5-HT2A receptor gene vary in terms of frequency, for example, Whites have six times more of the minor allelic version compared to Blacks. These and other findings add to evidence that the component is a receptor for chemical antidepressant action. Serotonergic genes have been also associated with suicide ideation, trauma in children, and criminality [24, 25].

Specifically related to chemical dependencies, these particular genes have been associated with heroin dependence. The 5-HT2A-1438A allele was significantly more common in heroin dependent patients than controls [0.55 and 0.45, respectively; corrected *P* = 0.042]. An interaction between A-1438G of 5-HT2A and 5-HTT polymorphisms was observed, in the presence of short 5-HTTLPR alleles and 12-repeat 5-HTT VNTR the association between heroin dependence and the −1438AA vs. AG/GG genotypes was enhanced [24.8 % in heroin-dependent patients vs. 12.6 % in controls; corrected *P* = 0.045] [26]. Moreover, genetic analyses showed that the frequency of 102C allele and C102C genotype in alcoholic subjects was significantly higher than in controls. In addition, alcoholic patients homozygous for C allele had alcoholic problems at significantly earlier age of onset than subjects having at least one T allele. These results point to the possibility of a role for the T102C HTR2A polymorphism in development of alcohol dependence and even relapse [27, 28]. Additionally, 5-HT2A, and 5-HT2C receptors that innervate the DA meso-accumbens pathway may play a prominent role in the behavioral effects of cocaine [29]. Smoking behavior is influenced by genetic factors affecting the dopaminergic system, and dopaminergic polymorphisms have been linked to smoking habits [30]. Since this T102C polymorphism of the 5-HT2A receptor gene modulates the mesolimbic DA system and is associated with reduced receptor gene expression, the purpose of one study [31] was to investigate the relationship it has to tobacco use. The T102C polymorphism was found to be associated with maintenance, but not with the initiation of the smoking habit. The CC genotype was more frequent in current smokers than in never- or former-smokers (chi2 = 6.825, *P* = 0.03) with an odds ratio of 1.63, 95 % CI 1.06–2.51. Interestingly, Nichols et al. [32] found that the gene response to LSD was quite dynamic. The expression of some genes increased rapidly and decreased rapidly while other genes changed more gradually. Dose-response studies showed two classes: (1) gene expression maximally stimulated at lower doses, and (2) gene expression that continued to rise at the higher doses. In a series of experiments that used receptor specific antagonists, the role of the 5-HT1A and 5-HT2A receptors in mediating the increases in gene expression, was examined and found that the 5-HT2A receptor activation was responsible for the majority of expression increases.

## 5-HTTLPR (Serotonin Transporter-Linked Polymorphic Region)

The human 5-HT transporter is encoded by the SLC6A4 gene on chromosome 17q11.1-q12. This is the site for cellular reuptake of 5-HT and a site where many drugs with central nervous system effects are activated. They include therapeutic agents like antidepressants and psychoactive drugs of abuse like cocaine. The 5-HT transporter has a prominent role in the metabolic cycle of many antidepressants, antipsychotics, anxiolytics, anti-emetics, and anti-migraine drugs. Higher expression of brain 5-HTT is associated with the (long allele) insertion variant compared to the (short allele) deletion variant. The results of some studies show that long allele is responsible for increased 5HT transporter mRNA transcription in human cell lines. Further, this may be due to the A-allele of rs25531, so that subjects with the long-rs25531 (A) allelic combination (LA) have higher levels, with the long-rs25531(G) earners have levels more similar to short-allele carriers. Saiz et al. [26] found an excess of -1438G and 5-HTTLPR L carriers in alcoholic patients in comparison to the heroin dependent group, the polymorphisms A-1438G and 5-HTTLPR also distinguished, alcohol from heroin dependent patients. The association of -1438A/G was especially pronounced with alcohol dependence when 5-HTTLPR S/S was present, less evident with 5-HTTLPR L/S, and not present with 5-HTTLPR.

The 5-HT transporter, encoded by the SLC6A4 gene, influences the synaptic actions of 5-HT and is responsive to stress hormones. In fact, the risk for suicidal behavior in CT exposed individuals is independently affected by the 5′ and 3′SLC6A4 functional variants [25]. National Longitudinal Study of Adolescent Health data shows that there is a significant gene-environment correlation between 5-HTTLPR and neglect for females only. Findings also reveal that 5-HTTLPR is associated with an increased risk of neglect for females and neglected females’ risk of abusing marijuana [33]. Socialization scores were significantly lower in males (greater sociopathy), with the L′L′ genotype (i.e., those homozygous for the L (A) allele) than males who carried the S′ allele (*P* = 0.03). In contrast, women with the S′S′ genotype tended to have a lower Socialization Index on the California Psychological Inventory than women with one copy of the L′ allele (*P* = 0.07) and lower socialization scores than women with two L′ alleles (*P* = 0.002). The tri-allelic 5-HTTLPR polymorphism had opposite effects on socialization scores in men than women with alcohol use disorders [34].

The genotype coding for low 5-HTT expression is associated with a better opioid analgesic effect, while the 5-HTTLPR s-allele has been associated with higher risk of developing chronic pain conditions. Downregulation of 5-HT1 receptors has been associated with the s-allele, and Kosek et al. [35] have suggested that individuals have an increased analgesic response to opioids during acute pain stimuli with a desensitization of 5-HT1 receptors, but may still be at increased risk of developing chronic pain conditions. The risk of alcohol dependence and co-occurring clinical features is increased in the presence of the short (S) allele of the 5-HT transporter gene promoter polymorphism (5-HTTLPR). While no other factor that were measured played a significant role, the S allele was significantly associated with relapse (*P* = 0.008). Thus, in abstinent alcohol-dependent patients the risk of relapse may be influenced by S allele of the 5-HTTLPR polymorphism, possibly through intermediate phenotypes [36].

## Catecholamine-*O*-Methyltransferase (COMT) Val158Met Polymorphism

Catechol-*O*-methyltransferase (COMT) was discovered in 1957 by the Nobel Prize Winner biochemist Julius Axelrod. COMT is an extra cellular enzyme that breaks down DA, adrenaline, and noradrenaline in the synapse. COMT is involved in the metabolism of the catecholamine neurotransmitters (DA, epinephrine, and norepinephrine). The enzyme introduces a methyl group donated by *SA*-denosyl-methionine to the catecholamine. Any compound having a catechol structure, like catechol-estrogens and catechol-containing flavonoids, are substrates of COMT, for example, l-dopa, a precursor of catecholamines and an important substrate of COMT. Variability of the COMT activity has previously been associated with the Val158Met polymorphism of the COMT gene and alcoholism. Serý et al. [37] found an association between alcoholism in male subjects and the Val158Met polymorphism of the COMT gene. Serý et al. also found the significant difference between allele and genotype frequencies of male alcoholics and male controls. In one of the subjects genotyped with heroin addiction, carries of the DRD2 A1 allele, also carried the low enzyme COMT activity genotype (A/A). This is in agreement with the work of Cao et al. [38] in 2003 who found no association with the high G/G and heroin addiction. No significant differences in genotype and allele frequencies of 108 val/met polymorphism of COMT gene were observed between heroin-dependent subjects and normal controls. No differences in genotype and allele frequencies of 900 Ins C/Del C polymorphism of COMT gene were observed between heroin-dependent subjects and normal controls. While there is still some controversy regarding the COMT association with heroin addiction, it was also interesting that the A allele of the val/met polymorphisms (−287 A/G) found by Cao et al. [38] was found to be much higher in heroin addicts than controls. Faster metabolism results in reduced DA availability at the synapse, which reduces postsynaptic activation, inducing hypodopaminergic functioning. Generally, Vandenbergh et al. [39] in 1997, and others [40] supported an association with the Val allele and Substance Use Disorder, but others did not [41]. Li et al. [42] found the COMT rs737866 gene variants were independently associated with both novelty seeking (NS) and age of onset of drug use. Those subjects with the TT genotype had higher NS subscale scores and an earlier onset age of heroin use than individuals with CT or CC genotypes. In a multivariate analysis, the inclusion of the NS sub score variable weakened the relationship between the COMT rs737866 TT genotype and an earlier age of onset of drug use. Li’s findings that COMT is associated with both NS personality traits and with the age of onset of heroin use helps to clarify the complex relationship between genetic and psychological factors in the development of substance abuse. Case-control analyses did not show any significant difference in allele or genotype distributions. However, a dimensional approach revealed a significant association between the COMT-Val (158) Met and NS. Both controls and opiate users with Met/Met genotypes showed higher NS scores compared to those with the Val allele. Demetrovics et al. [43] reported the NS scores also were significantly higher among opiate users; however, no interaction was found between group status and COMT genotype. A functional single nucleotide polymorphism (a common normal variant) of the gene for COMT has been shown to affect cognitive tasks broadly related to executive function, such asset shifting, response inhibition, abstract thought, and the acquisition of rule sorting structure. This polymorphism in the COMT gene results in the substitution of the amino acid valine for methionine. It has been shown that this valine variant catabolizes DA at up to four times the rate of its methionine counterpart resulting in a significant reduction of synaptic DA following neurotransmitter release, ultimately reducing dopaminergic stimulation of the post-synaptic neuron [44] another driver in the GARS.

## Monoamine Oxidase-A

Monoamine oxidase-A (MAOA) is an enzyme that degrades the neurotransmitters 5-HT, norepinephrine, and DA in the mitochondria. MAOA is involved with both physical and psychological functioning and classified as a flavoprotein since it contains the covalently bonded cofactor FAD. MAOA is an oxidative catalyst that uses oxygen to deaminate-remove an amine group from molecules, resulting in the corresponding aldehyde and ammonia.

Both forms of MOA (A and B) enzymes are substrates for the activity of a number of monoamine oxidase inhibitor drugs and are, therefore, well known in pharmacology. They are particularly important in the catabolism of monoamines ingested in food and vital to the inactivation of monoaminergic neurotransmitters. They display different specificities MAO-A primarily breaks down serotonin, melatonin, norepinephrine, and epinephrine while phenylalanine and benzyl amine are mainly broken down by MAO-B. Both forms break down DA, tyramine and tryptamine equally.

The gene that encodes MAOA is found on the X chromosome is located 1.2 kb upstream of the MAOA coding sequences and contains a polymorphism (MAOA-uVNTR) [45]. The MAOA-uVNTR consists of a 30-base pair repeated sequence, six allele variants containing either 2-, 3-, 3.5-, 4-, 5-, or 6-repeat copies [46]. Functional studies have indicated the alleles confer variations in transcriptional efficiency, for example, the 3.5- and 4-repeat alleles result in higher efficiency, whereas, the 3-repeat variant conveys lower efficiency [47]. To date, there are fewer consensuses regarding the transcriptional efficiency of the other less commonly occurring alleles, for example, 2-, 5-, and 6-repeat. The MAOA gene is a highly plausible candidate for effecting differences in the manifestation of psychological traits and psychiatric disorders based on its primary role in regulating monoamine turnover, and thereby influencing levels of norepinephrine, DA, and 5-HT [48]. Levels of MAO-A in the brain of patients with major depressive disorder, measured using positron emission tomography (PET), are elevated by an average of 34 %. Recently, evidence has indicated that the MAOA gene may associate with depression [49] and stress [50]. Evidence regarding whether lower or higher transcriptional efficiency of the MAOA gene, is positively associated with psychological pathology, has however, been mixed. The MAOA-uVNTR polymorphism low-activity 3-repeat allele has been positively related to symptoms of cluster B personality disorders and antisocial personality [51]. Other studies suggest that unhealthy psychological characteristics such as trait aggressiveness and impulsivity are related to alleles associated with higher transcriptional efficiency. Low MAO activity and the neurotransmitter DA are both important factors in the development of alcohol dependence. Huang et al. [52] investigated whether the association between the *DRD2* gene and alcoholism is affected by different polymorphisms of the MAO type A (*MAOA*) gene since MAO is an important enzyme associated with the metabolism of biogenic amines. They found that the genetic variant of the *DRD2* gene associated with the anxiety, depression (ANX/DEP) alcoholic phenotype, and the genetic variant of the MAOA gene was associated with alcoholism. Specifically, subjects carrying the MAOA 3-repeat allele and genotype A1/A1 of the DRD2 were 3.48 times more likely to be ANX/DEP alcoholics than the subjects carrying the MAOA 3-repeat allele and DRD2 A2/A2 genotype. Thus, the MAOA gene may modify the association between the DRD2 gene and ANX/DEP alcoholic phenotype. Overall, Vanyukov et al. [53] suggested that, although not definitive, variants in MAOA account for a small portion of the variance of risk for Substance Use Disorder, possibly mediated by liability to early onset behavioral problems.

## Dopamine D1 Receptor Gene

The DA receptor D1, also known as DRD1 a subtype of the DA receptor is a protein encoded by the DRD1 gene and the most abundant DA receptor in the human central nervous system where it expresses primarily in the caudate putamen. This G-protein-coupled receptor activates cyclic AMP-dependent protein kinases and stimulates adenylyl cyclase. D1 receptors regulate neuronal growth and development, modulate DA D2 receptor-mediated events and mediate some behavioral responses. There are two transcript variants of the DRD1 gene that are initiated at alternate transcription sites.

The DA D1 receptor has been associated with many brain functions that include, motor control, inattentive symptoms, and reward and reinforcement mechanisms. Betel et al. [54] found that the DRD1 gene polymorphism T allele of the rs686 was significantly (*P* = 0.0008) more frequent in patients with alcohol dependence. Frequency increased with severe dependence and was even higher for patients with severe complications like withdrawal seizures. Alcohol dependence was significantly, more precisely associated with a specific haplotype rs686*T-rs4532*G within the DRD1 gene. In another study, Kim et al. [55] found that the severity of the alcohol-related problem as measured by the Alcohol Use Disorders Identification Test in a gene dose-dependent manner, was significantly associated with one 5′ UTR polymorphism in the DRD1 (DRD1-48A>G) gene; 24.37 (±8.19) among patients with −48A/A genotype, 22.37 (±9.49) among those with −48A/G genotype, and 17.38 (±8.28) among those with −48G/G genotype (*P* = 0.002). Novelty seeking, harm avoidance and persistence were also found to be associated with the DRD1-48A>A genotype. Most recently, Peng et al. [56] indicated that DRD1 gene polymorphism may be associated with the rapid acquisition of heroin dependence, from first drug use but may not play an important role in the susceptibility to heroin dependence in the Chinese Han population. Others have also found significant associations with opiate abuse relative to controls [57]. DRD1 antagonists may indeed reduce the acquisition of cocaine-cue associations and cocaine-seeking behavior. Genetic association studies revealed that polymorphisms of the DRD1 gene significantly associated with nicotine dependence [58]. Ni et al. [59] found a significant association between DRD1 and bipolar disorder for haplotype TDT analysis. Thus, these results suggest DRD1 may play a role in the etiology of bipolar disorder.

## Dopamine D2 Receptor Gene (DRD2)

DA receptor D_2_, also known as DRD2, is a protein that is encoded by the *DRD2* gene which encodes the D_2_ subtype of the DA receptor in humans. This G protein-coupled receptor inhibits adenyl cyclase activity. Two transcript variants encode different isoforms and a third variant that has been described are the result of alternative splicing of the gene. In mice, of D2R surface expression is regulated in the dentate gyrus by the calcium sensor NCS-1 and controls exploration, synaptic plasticity, and memory formation.

DA has the chemical formula (C_6_H_3_ (OH)_2_-CH_2_-CH_2_-NH_2_) is a member of the catecholamine family. DA is a precursor to the chemical messengers’ epinephrine (adrenaline) and norepinephrine (noradrenaline). Arvid Carlsson won a share of the 2000 Nobel Prize in Physiology and Medicine for showing that DA is not just a precursor to these substances, but is also a neurotransmitter.

Older antipsychotic drugs, like haloperidol and chlorpromazine are DRD2 antagonists, however they are exceedingly nonselective, being selective for the “D2-like family” receptors; binding to D2, D3, and D4 and many other receptors, such as, those for 5-HT and histamine. This lack of selectivity makes them difficult to research and results in a range of side effects. Similarly, older DA agonists like bromocriptine and cabergoline used to treat Parkinson’s disease are also poorly selective. However, the number of selective D2 ligands available for scientific research is likely to increase.

Almost a decade before Carlsson and others were awarded the Nobel Prize the DA D2 receptor gene (DRD2) was first associated with severe alcoholism and is today the most widely studied gene in psychiatric genetics [7]. The *Taq*1 A is a SNP (rs: 1800497) originally thought to be located in the 3′-untranslated region of the DRD2 but has since been shown to be located within exon 8 of an adjacent gene, the ankyrin repeat and kinase domain containing 1 (ANKK1). Importantly, while there may be distinct differences in function, the miss-location of the *Taq*1A allele may be attributable to the ANKKI and the DRD2 being on the same haplotype or the ANKKI being involved in reward processing through a signal transduction pathway [60]. The ANKKI and the DRD2 gene polymorphisms may have distinct and different actions with regard to brain function [61]. Presence of the A1^+^ genotype (A1/A1, A1/A2) compared to the A^−^ genotype (A2/A2) is associated with reduced receptor density [62–64]. This reduction causes hypodopaminergic functioning in the DA reward pathway. Other DRD2 polymorphisms such as the C (57T, A SNP (rs: 6277)) at exon 7 also associates with a number of RDS behaviors including drug use [65]. Compared to the T^−^ genotype (C/C), the T^+^ genotype (T/T, T/C) is associated with reduced translation of DRD2 mRNA and diminished DRD2 mRNA, leading to reduced DRD2 density and a predisposition to alcohol dependence [66]. The *Taq*1 A allele is a predictive risk allele in families [67].

More recently, Kraschewski and colleagues [68] found the DRD2 haplotypes I-C-G-A2 and I-C-A-A1 to occur with a higher frequency in alcoholics. The rare haplotype I-C-A-A2 occurred less often in alcoholics and was less often transmitted from parents to their affected children (1 vs. 7). Among the subgroups, I-C-G-A2 and I-C-A-A1 had a higher frequency in Cloninger 1 alcoholics and alcoholics with a positive family history. Cloninger 2 alcoholics had a higher frequency of the rare haplotype D-T-A-A2 as compared with controls. In patients with a positive family history, haplotype I-C-A-A2 and Cloninger 1 alcoholics, haplotype I-T-A-A1 was less often present, confirming that haplotypes, which are supposed to induce a low DRD2 expression, were associated with alcohol dependence. Furthermore, supposedly high-expressing haplotypes weakened or neutralized the action of low-expressing haplotypes [68]. Moreover, Kazantseva et al. [69] found, significant effects of the ANKK1/DRD2 *Taq1*A on “Neuroticism” and of SLC6A3 rs27072 on “Persistence” in both genders. The association between ANKK1/DRD2 *Taq*1A A2/A2-genotype and higher Novelty Seeking and lower Reward Dependence was shown in men but not in women.

## Dopamine D3 Receptor Gene

The DA D3 receptor is a protein that is encoded by the DRD3 gene in humans and is a subtype of the DA receptor which inhibits adenylyl cyclase through inhibitory G-proteins. This receptor is expressed in older regions of the brain phylogenetically. This suggests that it is important in emotional functions. It is a target for drugs that treat Parkinson’s disease, schizophrenia, and drug addiction. Differently encoded isoforms from alternative gene splicing result in transcription of multiple variants. Some of these variants may be subject to nonsense-mediated decay, however, in rodent models of depression D3 agonists like pramipexole, rotigotine, and 7-OH DPAT, among others, display antidepressant effects.

Data from Vengeliene et al. [70] revealed an up-regulation of the DA D3 receptor (D3R) in the striatum after 1 year of voluntary alcohol consumption of alcohol preferring rats that was confirmed by qRT-polymerase chain reaction. This finding was further supported by up-regulation of striatal D3R mRNA found in non-selected Wistar rats, after long-term alcohol consumption when compared with age-matched control animals. Moreover, they examined the role of the D3R in mediating alcohol relapse behavior. They used the alcohol-deprivation-effect model, in long-term alcohol drinking Wistar rats and the model of cue-induced reinstatement of alcohol-seeking behavior, using the selective D3R antagonist SB-277011-A (0, 1, 3, and 10 mg/kg) and the partial agonist BP 897 (0, 0.1, 1, and 3 mg/kg). Both treatments caused a dose-dependent reduction of relapse-like drinking in the alcohol-deprivation-effect model, as well as a decrease in cue-induced ethanol-seeking behavior. They concluded that long-term alcohol consumption led to an up-regulation of the DA D3R that might have contributed to alcohol-seeking and relapse. Moreover, the Gly9, Gly9 genotype of the DRD3 Ser9Gly polymorphism was associated with increased rates of obsessive personality disorder symptomatology [71].

Furthermore, several lines of evidence indicate that dopaminergic neurotransmission is involved in the regulation of impulsive aggression and violence and that genetically determined variability in dopaminergic gene expression modifies complex traits including that of impulsivity and aggression. In one study, Retz et al. [72] reported an association of the DRD3 polymorphism with impulsiveness according to Eysenck’s EIQ and scores on the German short version of the Wender Utah Rating Scale (WURS-k), which they used for the assessment of a history of attention deficit hyperactivity disorder (ADHD) symptoms. This association was detected in a group of violent offenders, but not in non-violent individuals. Highest scores of EIQ impulsiveness and the WURS-k were found in heterozygous violent individuals while homozygotes showed significant lower rating scores, suggesting a heterosis effect. The results of their study suggest that variations of the DRD3 gene are likely involved in the regulation of impulsivity and some psychopathological aspects of ADHD related to violent behavior.

Finally, as opiates increase DA transmission, Spangler et al. [73] measured the effects of morphine on DA-related genes using a real-time optic PCR assay that reliably detects small differences in mRNA in discrete brain regions. As reported previously by others, there was no alteration in D1R mRNA and a 25 % decrease in D2R mRNA in the caudate-putamen, 2 h after the final morphine injection. Importantly, in the same RNA extracts, D3R mRNA showed significant increases of 85 % in the caudate-putamen and 165 % in the ventral midbrain, including the substantia nigra and VTA. There were no other significant morphine effects. The understanding of the ability of D3R agonists to reduce the effects of morphine was extended by the finding that chronic intermittent injections of morphine caused an increase in D3R mRNA extends.

## Dopamine D4 Receptor Gene

The DA receptor D4 is encoded by the DRD4 gene on chromosome 11 located in 11p15.5 in humans. Like the DRD2 the D4 receptor is a G protein-coupled receptor, activated by the neurotransmitter DA. It also inhibits the adenylate cyclase enzyme which reduces the intracellular second messenger cyclic AMP concentration. The DRD4 has been associated with numerous psychiatric and neurological conditions including addictive behaviors, and eating disorders (like binge-eating anorexia and bulimia nervosa, bipolar disorder) schizophrenia and Parkinson’s disease. Slight variations (mutations/polymorphisms) in the human DRD4 gene include: A 48-base pair VNTR in exon 3; 13-base pair deletion of bases 235 to 247 in exon 1; C-521T in the promoter; Val194gLY; 12 base pair repeat in exon I; A polymorphic tandem duplication of 120 bp. These mutations have been associated with a number of behavioral phenotypes, including autonomic nervous system dysfunction, ADHD, schizophrenia, and the personality trait of novelty seeking. Specifically, the 48-base pair VNTR in exon 3 ranges from 2 to 11 repeats. Polymorphisms with 6 to 10 repeats are the “Long” versions of the alleles. The frequency of the alleles varies considerably between populations, for example, the incidence of the 7-repeat version is high in America and low in Asia. The DRD4 long variant, or more specifically the 7 repeat (7R), has been loosely linked to psychological traits and disorders like susceptibility for developing ADHD appears to react less strongly to DA molecules. The 7R allele appears to have been selected about 40,000 years ago then in 1999, Chen et al. [74, 75] showed that nomadic populations had higher frequencies of 7R alleles than sedentary ones. They also observed that higher frequency of 7R/long alleles in populations who migrated further from 1,000 to 30,000 years ago. Recently, it was found that Ariaals with the 7R alleles, who are newly sedentary (non-nomadic), are not as healthy as nomadic Ariaal men with 7R alleles [76].

Despite early findings of an association between the DRD4 48 bp VNTR and novelty seeking (characteristic of exploratory and excitable people), a 2008 meta-analysis compared 36 published studies of novelty seeking and the polymorphism and found no effect. The meta-analysis of 11 studies did find that another polymorphism in the gene, the −521C/T, showed an association with novelty seeking [77]. In any case, novelty-seeking behavior probably is mediated by several genes, and the variance attributable to DRD4 by itself is not particularly large.

Several studies have suggested that parenting may affect the cognitive development of children with the 7-repeat allele of DRD4 [78]. Parenting that has maternal sensitivity, mindfulness, and autonomy-support at 15 months was found to alter children’s executive functions at 18 to 20 months [78]. Children with poorer quality parenting were more impulsive and sensation seeking than those with higher quality parenting [78]. Higher quality parenting was associated with better effortful control in 4-year-olds [78] and these effects are impacted by epigenetic markers on chromatin structures.

There is evidence that the length of the D4 DA receptor (DRD4) exon 3 variable number of tandem repeats (VNTR) affects DRD4 functioning by modulating the expression and efficiency of maturation of the receptor [79]. The 7R VNTR requires significantly higher amounts of DA to produce a response of the same magnitude as other size VNTRs [80], and this reduced sensitivity or DA resistance leads to hypodopaminergic functioning. Thus, 7R VNTR has been associated with substance-seeking behaviors [81, 82]. However, not all reports support this association. Biederman et al. [83] evaluating a number of putative risk alleles using survival analysis, revealed that by 25 years of age, 76 % of subjects with a DRD4 7R allele were estimated to have significantly more persistent ADHD compared with 66 % of subjects without the risk allele. In contrast, there were no significant associations between the course of ADHD and the DAT1 10-repeat allele and 5HTTLPR long allele. These findings suggested that the DRD4 7R allele, is associated with increased persistence in the course of ADHD. Moreover, Grzywacz et al. [84] evaluated the role of DRD4 exon 3 polymorphisms (48 bp VNTR) in the pathogenesis of alcoholism and found significant differences in the short alleles (2–5 VNTR) frequencies, between controls and patients with a history of delirium tremens and/or alcohol seizures. A trend also was observed in the higher frequency of short alleles amongst individuals with an early age of onset of alcoholism. The results of this study suggest that inherited short variants of DRD4 alleles (3R) may play a role in the pathogenesis of alcohol dependence and carriers of the 4R may have a protective effect for alcoholism risk behaviors. It is of further interest that work from Kotler et al. [85] in heroin addicts illustrated that central dopaminergic pathways figure prominently in drug-mediated reinforcement including novelty seeking, suggesting that DA receptors are likely candidates for association with substance abuse. These researchers showed that the 7R allele was significantly over-represented in the opioid-dependent cohort and conferred a relative risk of 2.46.

## Dopamine Transporter Gene (DAT1)

The DA transporter, also known as DA active transporter (DAT, SLC6A3), moves DA the neurotransmitter out of the synapse back into the cytosol via a membrane-spanning protein pump. From there, DA and norepinephrine are sequestered by other transporters into vesicles for later storage and release. DA reuptake through which DA is cleared from synapses primary by the mechanism of the DAT gene, although in the prefrontal cortex, there may be an exception, where evidence points to a larger role for the norepinephrine transporter [86, 87].

DAT is thought to be implicated in a number of DA-related disorders, including ADHD, bipolar disorder, clinical depression, and RDS. The gene that encodes the DAT protein is located on human chromosome 5, consists of 15 coding exons and is roughly 64 kbp long. Evidence for the associations between DAT- and DA-related disorders has come from a type of genetic polymorphism, known as a VNTR, in the DAT gene (DAT1), which influences the amount of protein expressed. DAT is an integral membrane protein that removes DA from the synaptic cleft and deposits it into surrounding cells, thus terminating the signal of the neurotransmitter. DA underlies several aspects of cognition, including reward, and DAT facilitates regulation of that signal [88, 89].

In the model for monoamine transporter function that is most widely accepted, before DA can bind sodium ions must bind to the DAT extracellular domain. Once DA binds to sodium ions change in the conformation of the protein allows both the DA and the sodium to unbind intracellularly [90]. Studies using electrophysiology and radioactive-labeled DA have confirmed that DA is transported across the neuronal membrane with sodium ions. The chlorine ions are required to prevent a buildup of positive charge. These studies have demonstrated that the direction and rate of transport, depends on the sodium gradient [77]. It is known that any resultant activity changes in membrane polarity will profoundly affect transport rates and depolarization will induce DA release [91].

Like the GABA transporter, the brain distribution of DAT is highest in the nigrostriatal, mesocortical, and mesolimbic pathways [92] and gene expression patterns in adult mouse show that expression is high in the substantia nigra pars compacta [93]. It has been confirmed that DAT in the mesocortical pathway was also found in the VTA. DAT is localized in the axon terminals of the striatum and shown experimentally to be co-localized with nigrostriatal terminals, tyrosine, and DA D2 receptors. These results suggest that striatal DA reuptake may occur and DA diffuses into the substantia nigra from the synaptic cleft. It appears that DAT is transported into the dendrites, where it can be found in pre- and postsynaptic active zones, smooth endoplasmic reticulum, and plasma membrane. These localizations suggest that intracellular and extracellular DA levels of nigral dendrites are modulated by DAT.

## Genetics and Regulation

The gene for DAT is located on chromosome 5p15, it is confirmed that the protein encoding for the gene is over 64 kb long and comprises 15 coding segments or exons [94]. The DAT gene has a VNTR at the intron 8 region and another at the 3′ end (rs28363170) [95]. VNTR differences have been shown to influence the basal level of expression of the transporter and associate with RDS behaviors, such as various addictions and other DA-related disorders [96]. Interestingly, Nurri, a nuclear receptor that regulates many DA-related genes, can bind the promoter region of this gene and subsequently induce expression [97]. In addition, the DAT promoter also may be the target of the transcription factor Sp-1. Kinases are essential for functional regulation of this protein. The rate at which DAT removes DA from the synapse affecting the quantitative amount of DA in the cell depends upon kinases. Understanding the molecular neurobiology of the DAT gene provides the basis for studies showing hyperactivity, severe cognitive deficits, and motor abnormalities of mice with no DA transporters [98]. Similarities of these effects to the symptoms of ADHD characteristics are striking. Differences in the functional differences, in VNTR, have been identified as risk factors for bipolar disorder [99] and ADHD [100]. Although controversial, data have emerged that suggest there also is an association with stronger alcohol withdrawal symptoms [101, 102]. On the other hand, non-smoking behavior and ease of quitting is associated with an allele of the DAT gene of the with normal protein levels [103]. Additionally, male adolescents in high-risk families, with an absence of maternal affection and a disengaged mother, who carry the 10-allele VNTR repeat, associate with a statistically significant affinity for antisocial peers [104]. Increased DAT activity is associated with clinical depression [105]. Decreased DAT levels of expression are associated with aging, and may underlie a mechanism that compensates for decreases in DA release as a person ages [106].

Cocaine reduces the rate of transport, blocking DAT by binding directly to the transporter [107]. In contrast, amphetamines trigger a signal cascade thought to involve PKC or MAPK that leads to the internalization of DAT molecules, which are normally expressed on the neuron’s surface [108]. Amphetamine on DAT also has a direct effect in the increased levels of secreted DA [109]. Lipophilic AMPH diffuses into the cytoplasm and the DA secretory vesicles disrupting the proton gradient established across the vesicle wall. This induces a leaky channel, and DA diffuses out into the cytoplasm. Additionally, AMPH causes a reversal of normal DA flow at the DAT. Instead of DA reuptake, in the presence of AMPH, a reversal in the mechanism of DAT occurs causing an outflow of DA released into the cytoplasm into the synaptic space changing it from a symporter to an antiporter-like functionality [110–112]. These mechanisms both result in less removal of DA from the synapse, increased signaling, which may underlie the pleasurable feelings elicited by these substances [113].

The DA transporter protein regulates DA-mediated neurotransmission by rapidly accumulating DA that has been released into the synapse [113]. Moreover, within 3 noncoding region of DAT1 lies a VNTR polymorphism [113]. There are two important alleles that may independently increase risk for RDS behaviors. The 9 repeat (9R) VNTR has been shown to influence gene expression and to augment transcription of the DA transporter protein [114]. Therefore, this results in an enhanced clearance of synaptic DA, yielding reduced levels of DA to activate postsynaptic neurons. Presence of the 9R VNTR has been linked to Substance Use Disorder [115], but not consistently [116]. Moreover, in terms of RDS behaviors, Cook et al. [117] was the first group that associated tandem repeats of the DAT gene in the literature. While there have been some inconsistencies associated with the earlier results, the evidence is mounting in favor of the view that the 10R allele of DAT is associated with high risk for ADHD in children and adults alike. Specifically, it was the non-additive association for the 10-repeat allele to be significant for hyperactivity-impulsivity symptoms, but exploratory analyses of the non-additive association of the 9-repeat allele of DAT1 with HI and oppositional defiant disorder symptoms also was significant. However, Volkow’s [118] group found that 12 months of methylphenidate treatment significantly increased striatal DA transporter availability in ADHD (caudate, putamen, and ventral striatum) by 24 %, whereas there were no changes in control subjects retested at the 12-month interval.

## Gamma Aminobutyric Acid (GABA) 1519T>CGABA(A)alpha6Gene

The role of GABA as the primary inhibitory neurotransmitter is the regulation of neuronal excitability throughout human central nervous system. First synthesized in 1883, as a plant and microbe metabolic product, it was not until 1950 that GABA was found to be an integral to brain functioning and directly responsible for muscle tone regulation [119].

GABA acts at the brains inhibitory synapses where it binds to specific transmembrane receptors. This binding in the plasma membrane of both pre- and postsynaptic neuronal processes opens the ion channels, discovered by Nobel Prize winner Erwin Neher in 1991. Ion channels allow chloride ions which are negatively charged into the cell or potassium ions which are positively charged to flow out of the cell. These actions change the transmembrane potential, causing hypo- or hyperpolarization. Polarization depends on the direction chloride flow. GABA is depolarizing (excitatory) when net chloride flows out of the cell and hyperpolarizing (inhibitory) when the net chloride flows into the cell. It is known that as the brain develops into adulthood the role of GABA changes from excitatory to inhibitory [120].

There are two known classes of GABA receptor: GABA_A_ receptor, which is part, of a ligand-gated ion complex, and GABA_B_ (G protein-coupled) metabotropic receptors that use intermediaries to open or close ion channels. GABAergic neurons that produce GABA have a mostly inhibitory action at receptors, although some GABAergic neurons, such as chandelier cells are able to excite their glutamatergic receptors or neurons counterparts [120–123].

In the metabolic pathway, called the GABA shunt, the enzyme l-glutamic acid decarboxylase and pyridoxal phosphate, the active form of vitamin B6, is used, as a cofactor, to synthesize GABA in the brain from glutamate, the principal excitatory neurotransmitter. In this process that converts glutamate into GABA [124, 125], the GABA transaminase enzyme catalyzes the conversion of 2-oxoglutarate and 4-aminobutanoic acid into glutamate and succinic semialdehyde. Then the enzyme succinic semialdehyde dehydrogenase oxidizes succinic semialdehyde into succinic acid which enters the citric acid cycle as a usable source of energy [126].

Drugs, known as GABA analogues or GABAergic drugs act as allosteric modulators of GABA receptors, to increase the amount of available GABA and usually have, anti-anxiety, anti-convulsive, and relaxing effects [127, 128]. In general, GABA does not cross the blood-brain barrier [129]. At least one study suggests that blocking GABA increases the amount of DA released [130].

## Genetics of GABA Receptor Gene Polymorphisms

GABA receptor genes have received some attention as candidates for drug use disorders. One reason for this is that the DA and GABA systems are functionally interrelated [112]. Research suggests that DA neurons projecting from the anterior VTA to the NAc are tonically inhibited by GABA through its actions at the GABAA receptor [131]. Moreover, it has been shown that alcohol [132] or opioids [133] enhancement of GABAergic (through GABAA receptor) transmission inhibits the release of DA in the mesocorticolimbic system. Thus, a hyperactive GABA system, by inhibiting DA release, could also lead to hypodopaminergic functioning. Because of this, GABA genes are of interest in the search for causes of drug use disorders. A dinucleotide repeat polymorphism of the GABA receptor β3 subunit gene (GABRB3) results in either the presence of the 181-bp G1 or 11 other repeats designated as non-G1 (NG1) [134]. Research indicates that the NG1 has an increased prevalence in children of alcoholics [134]. Presence of the NG1 has been associated with alcohol dependence [135, 136]. In addition, other GABA receptor genes have been associated with this disorder [137]. In fact, craving for alcohol and food has been studied in association with alcohol dependence and eating disorders, respectively. Thus, one subclass of the GABA receptor, 1519T>C GABA (A) alpha 6 has been associated with both ethanol dependence and weight gain. This gene polymorphism has been associated with hypo-cortisolism and perhaps abdominal obesity. Interestingly, the pathophysiology may involve various epigenetic factors, such as stress that destabilize the GABA-hypothalamic-pituitary adrenal systems in those with genetic vulnerability. In T-allele carriers, the change in craving for alcohol during treatment for alcohol dependence is negatively associated with changes in craving for food as well [137].

Expression patterns of GABAergic genes in rodent brains have been elucidated but not in humans. There are many GABAergic pathways and factor analysis involving global expression on 21 of these pathways has been performed. Specifically, postmortem data from hippocampus of eight alcoholics, eight cocaine addicts, and eight controls was evaluated to determine factor specificity for response to chronic alcohol/cocaine exposure. While RNA-Seq six gene expression factors were identified and loaded onto two factors, most genes loaded (≥0.5) onto one factor. These analyses led to the understanding that the chromosome 5 gene cluster was the largest factor (0.30 variance) and as such encodes the most common GABAA receptor, α1β2γ2, and genes encoding the α3β3γ2 receptor. Interestingly, within this factor, these genes were unresponsive to chronic alcohol/cocaine exposure. However, chronic alcohol/cocaine exposure affected chromosome 4 gene-cluster factor (0.14 variance) that encoded the α2β1γ1 receptor. Moreover, in both alcoholics and cocaine-dependent humans, two other factors (0.17 and 0.06 variance) included genes specifically involved in GABA synthesis and synaptic transport showed expression changes. It has been suggested that there is specificity of GABAergic gene groups, for response to chronic alcohol/cocaine exposure. Thus, understanding the nature of GABAergic gene group specificity could have therapeutic implications for combating stress-related craving and relapse [138].

## Mu-Opioid Receptor Genes

Opioid receptors are a group of G-protein coupled receptors with opioids as ligands [139–141]. The original work on these receptors occurred in the late 1960s, with the first group being that of Avram Goldstein at Stanford [142]. The endogenous opioids are dynorphins, enkephalin, endorphins, endomophins, and nociception. In 1973, Pert and Snyder were first to publish a detailed binding study of what turned out to be the mu-opioid receptor using 3H-naloxone [143], although two other studies followed shortly after [144, 145]. The opioid receptors are ~40 % identical to somatostatin receptors (SSTRs). Opiate receptors are found in the spinal cord digestive tract and are distributed widely in the brain. The mu opiate receptor (OPRM) has high affinity for enkephalin, beta endorphins, and morphine.

An International Union of Basic and Clinical Pharmacology (IUPHAR) subcommittee has recommended that appropriate terminology for the four principal subtypes of opioid receptors the three classical (μ, δ, κ) receptors, and the non-classical (nociception) receptor, should be MOP, DOP, KOP, and NOP, respectively [146–148] (Table 3).

**Table 3:** Four major subtypes of opiate receptors

Receptor	Subtypes	Location	Function
delta (δ) DOP OP_1_^(I)^	δ_1_, δ_2_	• Brain ▪ Pontine nuclei ▪ Amygdala ▪ Olfactory bulbs ▪ Deep cortex • Peripheral sensory neurons	Analgesia Antidepressant effects Convulsant effects Physical dependence Perhaps of mu-opioid receptor-mediated respiratory depression
kappa (κ) KOP OP_2_^(I)^	κ_1_, κ_2_, κ_3_	Brain • Hypothalamus • Periaqueductal gray • Claustrum Spinal cord • Substantia gelatinos • Peripheral sensory neurons	Analgesia Anticonvulsant effects Dissociative and delirium effects Diuresis Dysphoria Miosis Neuroprotection Sedation
mu (μ) MOP OP_3_^(I)^	μ_1_, μ_2_, μ_3_	Brain • Cortex (laminae III and IV) • Thalamus • Striosomes • Periaqueductal gray • Rostral ventromedial medulla Spinal cord • Substantia gelatinosa • Peripheral sensory neurons • Intestinal tract	μ_1_: • Analgesia • Physical dependence μ_2_: • Respiratory depression • Miosis • Euphoria • Reduced GI motility • Physical dependence μ_3_: • Possible vasodilation

^a^The name is based on order of discovery (from Wikipedia)

## Genetics of the Mu Opiate Receptor Gene

The activity at the micro-opioid receptor is central to both pain responses and opioid addiction. The opioidergic hypothesis suggests variations at the opioid receptor mu 1 (OPRM1) gene locus associate with opiate addiction. Several SNPs in exon I contained in the OPRM1 gene, which encodes for mu-opioid receptor. The polymerase chain reaction-restriction fragment length polymorphism method was used to genotype SNPs, A118G (rs 1799971) and C17T (rs 1799972) that have been associated with substance abuse. For the 118G allele, the opioid dependents (*n* = 126) had an approximately 2.5-fold higher frequency of 0.31 (odds ratio 3.501; CI(95 %) 2.212–5.555; *P* < 0.0001) while the control subjects (*n* = 156) showed a frequency of 0.12. For the C17T polymorphism, 0.83 seen in opioid dependents (*n* = 123; odds ratio of 0.555; CI(95 %) 0.264–1.147; *P* = 0.121) versus the controls (*n* = 57) showed a frequency of 0.89 for C allele. Thus, a significant association was observed between the 118G allele, and opioid dependence but no association was found with C17T polymorphism [149].

The effects of opiate drugs on pain experiences differ in humans. Recent twin studies documented individual differences, in several types of pain that are likely to be substantially determined by genetics. Genetic components to migraine pain susceptibility are documented in large twin studies, although in family studies have found substantial genetic heterogeneity in migraine. Humans μOR densities also differ. Both in vivo PET radioligand analyses and binding studies to postmortem brain samples suggest ranges of 30–50 %, or even larger, in individual differences in human μOR densities. Elucidation of the genetic basis for these differences based on receptor expression would advance substantially understanding of individual differences in and drug responses and nociceptive behaviors. The likelihood of high or low levels of expression in an individual can be predicted by information about μOR gene polymorphisms and may allow drug treatments to be individualized. These data could aid in optimization of dose ranges and selection of analgesic agents. Pain management for individuals with acute or long-term pain problems could be improved. These data could also add new therapeutic specificities and efficacies to this well-established opiate drug class, still a major weapon for amelioration of pain states [150].

Decreased analgesic effects of opioids are associated with the SNP 118A>G in the micro-1 opioid receptor gene (OPRM1) [151]. One recent study shows that postoperative pain response in heterozygous patients is affected by the OPRM1 118A>G polymorphism. The efficacy of the analgesic therapy for postoperative pain may be impaired compared to patients with wild-type genes [151]. In another study, Liu and Wang [152] reported that the allelic frequency of variant (118G) allele was 39.6 %, and the prevalence of OPRM1-118 AA, AG, and GG genotypes was 31.3 % (*n* = 30), 58.3 % (*n* = 56), and 10.4 % (*n* = 10), respectively. For all patients, pre-treatment to post-treatment pain judgments were reduced significantly. Patients with AA genotype had a better analgesic effect than those with G allele variants (AG or GG genotypes). Pre- and post-treatment pain judgments for patients with G allele variants were also reduced significantly. However, for patients with AA genotype, pre-treatment and post-treatment pain judgments were especially dramatic. The requirement for rescue analgesia also was highest for patients with G allele variants. Interestingly, Setiawan et al. [153] found that one etiological pathway to alcoholism may be influenced by the A118G substitution, for which naltrexone pharmacotherapy is effective. Moreover, studies by Kranzler et al. [154] showed a modest association between OPRM1 alleles and substance (alcohol, cocaine, or opioid) dependence.

Finally, we provide a selective sample of the relationship of certain reward genes and their respective risk alleles (see Tables 4, 5, 6, 7, 8, 9, 10, 11, and 12).

**Table 4:** Dopamine D2 receptor gene (a sampling)

Polymorphism (s)	Study findings	References	Comments
SNP rs: 1800497	TaqA1 allele associates with sever alcoholism	Blum et al. (1990)	First study to associate with alcoholism (called reward gene)
ANKKI -p.Glu713Lys	DRD2 Taq1A RFLP is a single nucleotide polymorphism (SNP) that causes an amino acid substitution within the 11th ankyrin repeat of ANKK1	Neville et al. (2004)	The ANKKI gene is a reflection of DRD2 A_1_ allele
SNP rs: 1800497	This SNP has been found to predict future RDS behaviors as high as 74 %	Blum et al. (1996)	Using Bayesian analysis
SNP rs: 1800497	Presence of the A1^+^ genotype (A1/A1, A1/A2) compared to the A^−^ genotype (A2/A2), is associated with reduced density	Noble et al. (1991)	This reduction causes hypodopaminergic functioning in the dopamine reward pathway
SNP rs: 6277 at exon 7	T^+^ allele associates with alcohol dependence	Hill et al. (2008)	Associates with drug seeking behavior and other RDS behaviors
SNP rs: 1800497	10 year follow up that carriers of the DRD2 A1 allele have a higher rate of mortality compared to carriers of the A2 allele in alcohol dependent individuals	Dahlgren et al. (2011)	Taq IA1allele and a substantially increased relapse rate
DRD2-haplotypes I-C-G-A2 and I-C-A-A1	Confirmed the hypothesis that haplotypes, which are supposed to induce a low DRD2 expression, are associated with alcohol dependence	Kraschewski et al. (2009)	High frequency of haplotype was associated with Cloninger Type 2 and family history of alcoholism
SNP rs: 1800497	Genotype analysis showed a significantly higher frequency for the TaqIA polymorphism among the addicts (69.9 %) compared to control subjects (42.6 %; Fisher’s exact χ(2), *P* < 0.05)	Teh et al. (2012)	The addicts had higher scores for novelty seeking (NS) and harm avoidance (HA) personality traits

**Table 5:** Dopamine D4 receptor gene (a sampling)

Polymorphism(s)	Study findings	References	Comments
DRD4 - The 7 repeat (7R) VNTR	The length of the D4 dopamine receptor (DRD4) exon 3 variable number of tandem repeats (VNTR) affects DRD4 functioning by modulating the expression and efficiency of maturation of the receptor	Van Tol (1998)	The 7 repeat (7R) VNTR requires significantly higher amounts of dopamine to produce a response of the same magnitude as other size VNTRs
120 bp duplication, -616C/G, and -521C/T	Strong finding of -120 bp duplication allele frequencies with schizophrenia (*P* = 0.008); -521 C/T polymorphism is associated with heroin addiction	Lai et al.(2010)	This reduced sensitivity or “dopamine resistance” leads to hypodopaminergic functioning. Thus 7R VNTR has been associated with substance-seeking behavior
DRD4 7-repeat allele	A number of putative risk alleles using survival analysis revealed that by 25 years of age 76 % of subjects with a DRD4 7-repeat allele were estimated to have significantly more persistent ADHD compared with 66 % of subjects without the risk allele	Biederman et al. (2009)	Findings suggest that the DRD4 7-repeat allele is associated with a more persistent course of ADHD
7-repeat allele of the dopamine D(4) receptor gene (DRD4)	Although the association between ADHD and DRD4 is small, these results suggest that it is real	Faraone et al. (2001)	For both the case-control and family-based studies, the authors found (1) support for the association between ADHD and DRD4, (2) no evidence that this association was accounted for by any one study, and (3) no evidence for publication bias
dopamine D4 receptor (DRD4) exon 3 polymorphisms (48 bp VNTR)	Found significant differences in the short alleles (2–5 VNTR) frequencies between controls and patients with a history of delirium tremens and/or alcohol seizures (*P* = 0.043)	Grzywacz et al. (2008)	A trend was also observed in the higher frequency of short alleles amongst individuals with an early age of onset of alcoholism (*P* = 0.063)
dopamine D4 receptor (DRD4) -7 repeat allele	Show 7-repeat allele is significantly over-represented in the opioid-dependent cohort and confers 2.46 RR	Kotler et al. (1997)	First report of an association between opioid addiction and a genetic polymorphism

**Table 6:** Dopamine transporter gene (DAT1)

Polymorphism	Study findings	References	Comments
Localized to chromosome 5p15.3. Moreover, within 3 noncoding region of DAT1 lies a VNTR polymorphism -9 repeat (9R) VNTR	The 9 repeat (9R) VNTR has been shown to influence gene expression and to augment transcription of the dopamine transporter protein	Byerly et al. (1993)	Having this variant results in an enhanced clearance of synaptic dopamine, yielding reduced levels of dopamine to activate postsynaptic neurons
9 repeat (9R) VNTR	DAT1, genotype 9/9 was associated with early opiate addiction	Galeeva et al. (2002)	The combination of SERT genotype 10/10 with DAT1 genotype 10/10 was shown to be a risk factor of opiate abuse less than 16 years of age
exon 15 rs27072 and VNTR (DAT), promoter VNTR and rs25531	The haplogenotypes 6-A-10/6-G-10 and 5-G-9/5-G-9 were more often present in type 2 alcoholics as compared with type 1 alcoholics [odds ratio (OR): 2.8], and controls (OR: 5.8), respectively	Reese et al. (2010)	In a typology proposed by Cloninger on the basis of adoption studies, a subgroup has been classified as type2 with patients having high genetic loading for alcoholism, an early onset of alcoholism, a severe course, and coexisting psychiatric problems consisting of aggressive tendencies or criminality
VNTR polymorphism at the dopamine transporter locus (DAT1) 480-bp DAT1 allele	Using the haplotype-based haplotype relative risk (HHRR) method revealed significant association between ADHD/UADD and the 480-bp DAT1 allele (chi 2 7.51, 1 df, *P* = 0.006)	Cook et al. (1995)	While there have been some inconsistencies associated with the earlier results the evidence is mounting in favor of the view that the 10R allele of DAT is associated with high risk for ADHD in children and in adults alike

**Table 7:** Catechol-O-methyl-transferase (COMT) [a sampling]

Polymorphism(s)	Study findings	References	Comments
COMT Val 158 Met variation and DRD2 Taq1A genotypes	COMT Val158Met and DRD2 Taq1A may affect the intermediate phenotype of central dopamine receptor sensitivity	Schellekens et al. (2012)	COMT Val158Met and DRD2 Taq1A may confer their risk of alcohol dependence through reduced dopamine receptor sensitivity in the prefrontal cortex and hindbrain, respectively
The functional polymorphism (COMT Val108/158Met) affects COMT activity, with the valine (Val) variant associated with higher and the methionine (Met) variant with lower COMT activity	Male alcoholic suicide attempters, compared to male non-attempters, had the higher frequency of Met/Met genotype or Met allele, and significantly (Kruskal-Wallis ANOVA on ranks and Mann-Whitney test) higher aggression and depression scores	Nedic et al. (2011)	These results confirmed the associations between Met allele and aggressive behavior or violent suicide attempts in various psychiatric diagnoses, and suggested that Met allele of the COMT Val108/158 Met might be used as an independent biomarker of suicidal behavior across different psychopathologies
COMT Val(15) Met variation	Both controls and opiate users with Met/Met genotypes showed higher NS scores compared to those with the Val allele	Demetrovis et al. (2010)	Association of the COMT polymorphism and NS temperament scale has been shown for heroin-dependent patients and controls regardless of group status
A functional polymorphism (COMT Val158Met) resulting in increased enzyme activity has been associated with polysubstance abuse and addiction to heroin and meth-amphetamine	These results suggest a significant association between COMT Val158Met polymorphism and susceptibility to cannabis dependence	Baranse et al. (2008)	Cannabis stimulates dopamine release and activates dopaminergic reward neurons in central pathways that lead to enhanced dependence. Catechol-O-methyl transferase (COMT) inactivates amplified extraneuronally released dopamine

**Table 8:** Serotonin transporter gene (a sampling)

Polymorphism(s)	Study findings	References	Comments
Serotonin transporter promoter polymorphism [5-HT transporter gene-linked polymorphic region (5-HTTLPR)]	5-HTTLPR had age-dependent effects on alcohol, tobacco and drug use: substance use did not differ by genotype at age 9, but at age 15, the participants with the short (s)/s genotype had higher tobacco use, and at age 18, they were more active alcohol, drug and tobacco users	Merenäkk et al. (2011)	Results reveal that expression of genetic vulnerability for substance use in children and adolescents may depend on age, gender, interaction of genes, and type of substance
The short (s), low activity allele of a polymorphism (5-HTTLPR) in the serotonin transporter gene (SLC6A4) has been related to alcohol dependence.	The 5-HTTLPR short allele predicted adolescent’s growth (slope) in alcohol use over time. Adolescents with the 5-HTTLPR short allele showed larger increase in alcohol consumption than those without the 5-HTTLPR short allele	van der Zwaluw et al. (2010)	5-HTTLPR genotype was not related to the initial level (intercept) of alcohol consumption
triallelic 5-HTTLPR genotype : SA/SA and SA/LG compared to LA/LA	triallelic 5-HTTLPR genotype : SA/SA and SA/LG compared to LA/LA	Kosek et al. (2009)	Previously the 5-HTTLPR s-allele has been associated with higher risk of developing chronic pain conditions but in this study we show that the genotype coding for low 5-HTT expression is associated with a better analgesic effect of an opioid. The s-allele has been associated with downregulation of 5-HT1 receptors and we suggest that individuals with a desensitization of 5-HT1 receptors have an increased analgesic response to opioids during acute pain stimuli, but may still be at increased risk of developing chronic pain conditions.

**Table 9:** Mu opiate receptor (MOR) [a sampling]

Polymorphism(s)	Study findings	References	Comments
A single nucleotide polymorphism (SNP) in the human MOR gene (OPRM1 A118G) has been shown to alter receptor protein level in preclinical models and smoking behavior in humans	Independent of session, smokers homozygous for the wild-type OPRM1 A allele exhibited significantly higher levels of MOR BP (ND) than smokers carrying the G allele in bilateral amygdala, left thalamus, and left anterior cingulate cortex	Ray et al. (2011)	Among G allele carriers, the extent of subjective reward difference (de-nicotinized versus nicotine cigarette) was associated significantly with MOR BP(ND) difference in right amygdala, caudate, anterior cingulate cortex, and thalamus
Polymorphism in A118G in exon 1 and C1031G in intron 2 of the MOR gene	Results showed a significant association for both A118G and C1031G polymorphisms and opioid dependence. The G allele is more common in the heroin-dependent group (39.5 % and 30.8 % for A118G and C1031G polymorphisms, respectively) when compared to the controls (29.4 % and 21.1 % for A118G and C1031G polymorphisms, respectively)	Szeto et al. (2001)	This study suggests that the variant G allele of both A118G and C1031G polymorphisms may contribute to the vulnerability to heroin dependence
A118G single-nucleotide polymorphism (SNP) in exon 1 of the MOR gene (OPRM1), which encodes an amino-acid substitution, is functional and receptors encoded by the variant 118G allele bind the endogenous opioid peptide beta-endorphin with three-fold greater affinity than prototype receptors. Other groups subsequently reported that this variant alters stress-responsivity in normal volunteers and also increases the therapeutic response to naltrexone (a mu-preferring opioid antagonist) in the treatment of alcohol dependence	There was a significant overall association between genotypes with an 118G allele and alcohol dependence (*P* = 0.0074). The attributable risk for alcohol dependence in subjects with an 118G allele was 11.1 %	Bart et al. (2005)	There was no difference in A118G genotype between type 1 and type 2 alcoholics. In central Sweden, the functional variant 118G allele in exon 1 of OPRM1 is associated with an increased attributable risk for alcohol dependence
MOR gene knockout (KO) were examined in wild-type (+/+), heterozygote MOR KO (+/−) and homozygote MOR KO (−/−) mice on voluntary ethanol consumption	Heterozygous and homozygous MOR KO mice consumed less ethanol than wild-type mice. These effects appeared to be greater in female KO mice than in male KO mice. MOR KO mice, especially females, exhibited less ethanol reward in a conditioned place preference paradigm	Hall et al. (2001)	These data fit with the reported therapeutic efficacy of MOR antagonists in the treatment of human alcoholism. Allelic variants that confer differing levels of MOR expression could provide different degrees of risk for alcoholism

**Table 10:** GABA beta subunit 3 (a sampling)

Polymorphism(s)	Study findings	References	Comments
GABA A receptor beta3 subunit gene (GABRB3)	The G1- alleles of the GABRB3 in COAs were significantly higher than non COAs	Namkoong et al. (2008)	In the same study the frequency of the A1+ allele at DRD2 in the COAs was significantly higher than non COAs
beta 3 subunit m RNAs	The levels of the beta 2 and beta 3 subunit mRNAs remains elevated at 24 h withdrawal from chronic ethanol. Chronic ethanol treatment increased the levels of both of these polypeptides in cerebral cortex	Mhatre and Ticku (1994)	Chronic ethanol administration produced an up-regulation of the beta-subunit mRNA and the polypeptide expression of these subunits in rat cerebral cortex
A1+ (A1A1 and A1A2 genotypes) and A1− (A2A2 genotype) alleles of the DRD2 and G1+ (G1G1 and G1 non-G1 genotypes) and G1− (non-G1 non-G1 genotype) alleles of the GABRB3 gene,Study involved Mood-related alcohol expectancy (AE) and drinking refusal self-efficacy (DRSE) were assessed using the Drinking Expectancy Profile.	Patients with the DRD2 A1+ allele, compared with those with the DRD2 A1− allele, reported significantly lower DRSE in situations of social pressure. Similarly, lower DRSE was reported under social pressure by patients with the GABRB3 G1+ allele when compared to those with the GABRB3 G1− alleles. Patients with the GABRB3 G1+ allele also revealed reduced DRSE in situations characterized by negative affect than those with the GABRB3 G1− alleles. Patients carrying the GABRB3 G1+ allele showed stronger AE relating to negative affective change (for example, increased depression) than their GABRB3 G1− counterparts	Young et al. (2004)	Molecular genetics research has identified promising markers of alcohol dependence, including alleles of the D2 dopamine receptor (DRD2) and the GABAA receptor beta3 subunit (GABRB3) genes
Dinucleotide repeat polymorphisms of the GABA(A) receptor beta 3 subunit gene were compared to scores on the General Health Questionnaire-28 (GHQ)	Analysis of GHQ subscale scores showed that heterozygotes compared to the combined homozygotes had higher scores on the somatic symptoms (*P* = 0.006), anxiety/insomnia (*P* = 0.003), social dysfunction (*P* = 0.054) and depression (*P* = 0.004) subscales	Feusner et al. (2001)	The study indicates that in a population of PTSD patients, heterozygosity of the GABRB3 major (G1) allele confers higher levels of somatic symptoms, anxiety/insomnia, social dysfunction and depression than found in homozygosity
GABRB3 major (G1) allele and DRD@ A1 allele	A significant progressive increase was observed in DRD2 A1 allelic prevalence (*P* = 3.1 × 10(−6)) and frequency (*P* = 2.7 × 10(−6)) in the order of non-alcoholics, less severe and severe alcoholics. In severe alcoholics, compared to non-alcoholics, a significant decrease was found in the prevalence (*P* = 4.5 × 10(−3)) and frequency (*P* = 2.7 × 10(−2)) of the GABRB3 major (G1) allele. Furthermore, a significant progressive decrease was noted in G1 allelic prevalence (*P* = 2.4 × 10(−3)) and frequency (*P* = 1.9 × 10(−2)) in non-alcoholics, less severe and severe alcoholics, respectively	Noble et al. (1988)	In sum, in the same population of non-alcoholics and alcoholics studied, variants of both the DRD2 and GABRB3 genes independently contribute to the risk for alcoholism, with the DRD2 variants revealing a stronger effect than the GABRB3 variants. However, when the DRD2 and the GABRB3 variants are combined, the risk for alcoholism is more robust than when these variants are considered separately

**Table 11:** MOA-A (a sampling)

Polymorphism(s)	Study findings	References	Comments
MAOA genotype	Significant three-way interactions, MAOA genotype by abuse by sex, predicted dysthymic symptoms. Low-activity MAOA genotype buffered against symptoms of dysthymia in physically abused and multiply-maltreated women. Significant three-way interactions, MAOA genotype by sexual abuse by race, predicted all outcomes. Low-activity MAOA genotype buffered against symptoms of dysthymia, major depressive disorder, and alcohol abuse for sexually abused white participants. The high-activity genotype was protective in the nonwhite sexually abused group	Nikulina et al. (2012)	This prospective study provides evidence that MAOA interacts with child maltreatment to predict mental health outcomes
low-repeat MAOA allele	Individuals with CUD had reductions in GMV in the orbitofrontal, dorsolateral prefrontal, and temporal cortex and the hippocampus compared with controls. (2) The orbitofrontal cortex reductions were uniquely driven by CUD with low- MAOA genotype and by lifetime cocaine use	Alia-Klein et al. (2011)	Long-term cocaine users with the low-repeat MAOA allele have enhanced sensitivity to gray matter loss, specifically in the orbitofrontal cortex, indicating that this genotype may exacerbate the deleterious effects of cocaine in the brain
MAOA u-VNTR	Girls, carrying the long MAOA u-VNTR variant showed a higher risk of being high alcohol consumers, whereas among boys, the short allele was related to higher alcohol consumption	Nilsson et al. (2011)	The present study supports the hypothesis that there is a relation between MAOA u-VNTR and alcohol consumption and that this relation is modulated by environmental factors
30-bp repeat in the promoter region of the monoamine oxidase-A gene (MAO-A)	Significant associations between cold pain tolerance and DAT-1 (*P* = 0.008) and MAO-A (*P* = 0.024) polymorphisms were found. Specifically, tolerance was shorter for carriers of allele 10 and the rarer allele 11, as compared to homozygous for allele 9, and for carriers of allele 4 (MOA) as compared to homozygous for allele 3, respectively	Treister et al. (2009)	These results, together with the known function of the investigated candidate gene polymorphisms, suggest that low dopaminergic activity can be associated with high pain sensitivity and vice versa
The Revised Psychopathy Checklist (PCL-R) has shown a moderate association with violence and as such studied with MAOA genotyped alcoholic offenders	The PCL-R total score predicts impulsive reconvictions among high-activity MAOA offenders (6.8 % risk increase for every one-point increase in PCL-R total score, *P* = 0.015), but not among low-activity MAOA offenders, whereas antisocial behavior and attitudes predicted reconvictions in both genotypes (17 % risk increase among high-activity MAOA offenders and 12.8 % increase among low-activity MAOA offenders for every one-point increase in factor 2 score)	Tikkanen et al. (2011)	Results suggest that the efficacy of PCL-R is altered by MAOA genotype, alcohol exposure, and age, which seems important to note when PCL-R is used for risk assessments that will have legal or costly preventive work consequences
Genotyping of two functional polymorphisms in the promoter region of the serotonin transporter and monoamine oxidase-A, respectively, (5-HTT-LPR and MAOA-VNTR), was performed in a group of women with severe alcohol addiction.	Within the group of alcoholics, when the patients with known co-morbid psychiatric disorders were excluded, aggressive anti-social behavior was significantly linked to the presence of the high activity MAOA allele	Gokturk et al. (2008)	The pattern of associations between genotypes of 5-HTT-LPR and MAOA-VNTR in women with severe alcoholism differs from most corresponding studies on males
The MAOA gene presents several polymorphisms including a 30-bp VNTR in the promoter region (MAOA-uVNTR). Alleles with 3.5 and 4 repeats are 2–10 times more efficient than 3-repeat allele.	The results suggest that the 3-repeat allele is associated to: (1) alcohol dependence (*P* < 0.05); (2) an earlier onset of alcoholism (*P* < 0.01); (3) comorbid drug abuse among alcoholics (*P* < 0.05); and (4) a higher number of antisocial symptoms (*P* < 0.02)	Contini et al. (2006)	Results confirmed reports showing an association of the low activity 3-repeat allele of MAOA uVNTR polymorphism with substance dependence and impulsive, antisocial behaviors. These findings in a different culture support influence of MAOA-u VNTR in psychiatric illness

**Table 12:** Dopamine D3 (a sampling)

Polymorphism(s)	Study findings	References	Comments
The genotypes of the BDNF Val66Met and DRD3 Ser9Gly polymorphisms. BDNF regulates expression of D3.	Logistic regression analysis showed a significant main effect for the Val/Val genotype of the BDNF Val66Met polymorphism (*P* = 0.020), which predicted bipolar-II patients. Significant interaction effects for the BDNF Val66Met Val/Val genotype and both DRD3 Ser9Gly Ser/Ser and Ser/Gly genotypes were found only in bipolar-II patients (*P* = 0.027 and 0.006, respectively)	Lee et al. (2012)	Evidence that the BDNF Val66Met and DRD3 Ser9Gly genotypes interact only in bipolar-II disorder (hypomania) and that bipolar-I (Mania) and bipolar-II may be genetically distinct
D3R KO mice.	The possible interaction between morphine-induced tolerance and D3 receptors has not been investigated. Compared with wild-type (WT) mice, the dopamine D3 receptor knockout (D3R KO) mice showed pronounced hypoalgesia. The D3R KO mice clearly developed lower morphine-induced tolerance and showed attenuated withdrawal signs compared with the WT mice	Li et al. (2012)	These results suggest that D3 receptors regulate basal nociception and are involved in the development of morphine-induced tolerance and withdrawal
DNA microarrays of two different alcohol-preferring rat lines (HAD and P) and D3 receptors.	Data revealed an up-regulation of the dopamine D3 receptor (D3R) after 1 year of voluntary alcohol consumption in the striatum of alcohol preferring rats that was confirmed by qRT-polymerase chain reaction	Vengeliene et al. (2006)	Long-term alcohol consumption leads to an up-regulation of the dopamine D3R that may contribute to alcohol-seeking and relapse. We therefore suggest that selective antagonists of this pharmacological target provide a specific treatment approach to reduce alcohol craving and relapse behavior
Gly9 homozygotes in comparison to Ser9 carriers of D3 receptor gene.	German descent and have found diminished parietal and increased frontal P300 amplitudes in Gly9 homozygotes in comparison to Ser9 carriers. Further studies should address the direct role of the DRD3 Ser9Gly polymorphism in attenuated P300 amplitudes in psychiatric disorders like schizophrenia or alcoholism	Mulert et al. (2006)	An important reason for the interest in P300 event-related potentials are findings in patients with psychiatric disorders like schizophrenia or alcoholism in which attenuations of the P300 amplitude are common findings
Dopamine receptor D3 gene BalI polymorphism.	Patients above the median value for cognitive impulsiveness (one of the three dimensions of the Barratt scale) were more frequently heterozygous than both alcohol-dependent patients with lower impulsiveness (OR = 2.51, *P* = 0.019) and than 71 healthy controls (OR = 2.32, *P* = 0.025)	Limosin et al. (2005)	The D3 Receptor gene has been associated with addictive behaviors especially impulsiveness
Bal I polymorphism at the DRD3 gene	Patients with a sensation-seeking score above 24 were more frequently homozygotes for both alleles than patients with a sensation-seeking score under 24 (*P* = 0.038) or controls (*P* = 0.034)	Duaux et al. (1998)	These results suggest that the DRD3 gene may have a role in drug dependence susceptibility in individuals with high sensation-seeking scores
mRNA of both DRD2 and DRD3 gene expression	After a chronic schedule of intermittent bingeing on a sucrose solution, mRNA levels for the D2 dopamine receptor, and the preproenkephalin and preprotachykinin genes were decreased in dopamine-receptive regions of the forebrain, while D3 dopamine receptor mRNA was increased. The effects of sugar on mRNA levels were of greater magnitude in the nucleus accumbens than in the caudate-putamen	Spangler et al. (2004)	Striatal regions of sugar-dependent rats show alterations in dopamine and opioid mRNA levels similar to morphine-dependent rats

## Genetic Addiction Risk Score Panel

We are proposing a number of well- known risk alleles based on a plethora of literature based studies. Table 13 represents a list of each top gene (s) that should drive the risk stratification of the individual.

**Table 13:** GARS panel

Gene	Allele	Prime function
Dopamine D1 Receptor	48G	Regulation of dopamine release in accumbens
Dopamine D2 Receptor (ANKKI/DRD2)	Taq I A1	Controls synthesis of dopamine D2 receptors
Dopamine D3 Receptor (DRD3)	C	Carriers sensitive to cocaine; opioids, alcohol and nicotine
Dopamine D4 Receptor (DRD4)	7R	Pre-disposed to novelty seeking and ADHD
Dopamine Transporter (DAT1)	9R	Fast transport of synaptic dopamine back into pre-neuron leading to hypodopaminergic trait.
Serotonin Transporter (HTTLPR)	S	Fast transport of serotonin back into neuron
Mu-opiate Receptor (OPRM1)	G	Predisposes to heroin addiction and pain sensitivity
GABA B_3_ Receptor (GABAR3)	181.	Predisposes to anxiety disorders
Mono-Amine Oxidase A (MAO-uVNTR)	4R	Fast catabolism of mitochondria dopamine
Catecholamine Methyl-Transferase (COMT-vall58met)	G	Val substitution leads to fast catabolism of synaptic dopamine leading to RDS

Understanding that RDS is a very complex polygenic disorder, we have carefully considered and chosen the primary risk alleles based on thousands of support articles in the literature. We feel confident that GARS will provide a snapshot into RDS risk. We are cognizant that this panel may change over time as new and other gene polymorphisms are discovered. Moreover, until we can develop a weighted algorithm based on utilization of “super controls” for RDS, we cannot provide a perfect test.

## GWAS vs Candidate Gene Approach Issues

The need for genetic testing as a way of understanding or pinpointing therapeutic targets is certainly the wave of the future. Since the earliest study of the DRD2 gene, a remarkable list of associations with gene polymorphisms has been elucidated and eventually morphed into in the field, known today as Psychiatric Genetics. The DSM criteria are not the only method of diagnosis of psychiatric disorders. We are proposing that coupling verified standard pencil and paper tests, like the Addiction Severity Index among others, with DSM and GARS should enhance the understanding of each patient presenting for addiction treatment. In fact, it will remove guessing by providing objective risk stratification, as well as opportunities for DNA-targeted therapies (personalized addiction medicine). We are beginning to understand the power of Genome Wide Association (GWAS) [155] and EWAS studies, especially the epigenetics of gene expression via mRNA transcription. This knowledge could pave the way for either nutraceutical nutrigenomic solutions or highly specific pharmaceuticals, that by targeting select neuronal sites can reduce toxic side effects. In either case, we strongly recommend additional studies to provide the recovering addict with an epigenetic [156–158] tool to activate DA D2 receptors while attenuating the anti-reward effects of DA D1 and possibly D3 receptors, respectively. Finally, we as neuroscientists should begin to perform studies that control for possible comorbid medical and psychiatric conditions in the research (dual diagnosis). This work supports earlier non-genetic concepts of addiction in psychiatric medicine [159–163].

We are cognizant that there is controversy related to the importance of both GWAS and Whole-Exome Sequencing (WES) and analytical approaches relative to candidate association and linkage studies, used to unravel the contribution of specific genes and associated polymorphisms to addiction liability. In the late 1980s realizing the complexity of the problem of both vulnerability and resilience for risk of drug abuse and other behavioral addictions, we decided to analyze candidate genes based on a theoretical model developed and subsequently published, identified as the “Brain Reward Cascade” [1]. Initially our approach utilizing this “blue print of reward” involved association rather than linkage analysis because Lander et al. argued against linkage analysis for complex disorders like drug addiction [8] instead of linkage analysis as previously accomplished with one gene one disease (OGOD).

Important advances have been made over the last two decades concerning “Psychiatric Genetics”. Certainly, substantial genetic contributions to addiction liability are now supported by earlier twin studies and more recently linkage, candidate association and GWAS studies. Animal studies initially focusing upon genes that targeted mechanism of action of major drugs of abuse. Many of these studies were successful in the identification of quantitative trait loci including association of chromosomal 9 (DRD2 gene) and ethanol behavioral responses [164, 165] as well as other reward genes including serotonin, opioids and GABA [166]. Most of these and many other studies have identified gene/proteins that affect responses to drugs of abuse. Parallel to these animal studies, human genetic research has evolved over the last 5 years. Now, it is possible to detect genetic variation in the human genome inexpensively. The era of genome sequencing began with the detection of SNPs on gene chips. Very recently, we are also utilizing WES, which is a high-throughput sequencing, to identify the molecular arrangement of DNA base pairs specifying the coding regions of a person’s genome also referred to as the exome. While this is exciting, it may not have clinical utility because the exome only comprises about 1 % of the entire genome [167].

When compared to the enormous literature on candidate gene analysis (6120 studies) currently there is a paucity of GWAS/WES studies relevant to addiction liability (239 studies). A commentary by Hall et al. [168] from NIDA and others have argued that candidate gene analysis may be wrong. However, they do suggest the following from many GWAS studies: (1) addiction is highly polygenic, each allelic variant contributing in a small, additive (counting) amount to addiction liability; (2) classes of genes (such as reward circuitry based genes) are most important in explaining both risk and resilience to all addictions and (3) substantial genetic heterogeneity exists, and there is a convergence of GWAS signals on particular candidate-genes.

It is well-known that the action of psychoactive drugs primarily affects synaptic neurotransmission. Reynolds et al. [169] correctly suggest that specific genes for neurotransmitter receptors and transporters have provided strong candidates in pharmacogenetic research in psychiatry. Moreover, there are many inconsistencies between candidate gene and GWAS studies. Reynolds further suggests that consistencies have accumulated through candidate gene studies involving the dopamine D2 receptor; serotonin transporter; and GABA dysfunction in mental illness. Moreover, Han et al. [170] reported that following GWAS, functional enrichment analysis revealed specific genes to underlie alcohol risk such as cation transport, synaptic transmission, and transmission of nerve impulses representing meaningful biological processes. In agreement, Uhl et al. [171] suggest that following GWAS, clusters of SNPs within selected genes display 10(−2) > *P* > 10 (−8) associations with dependence in many independent studies. Importantly, along these lines, specific candidate genes associated with substance dependence phenotypes, for example, in Native Americans, these include *OPRM1*, *CRN1*, *COMT*, *GABA2*, *MAOA*, and *HTR3-B* [172]. In a cross-species GWAS study to access risk genes, in alcoholism, it was found that 47 genes associated including GABA, signaling pathway and cell communication [173]. In one study using GWAS and factor analysis, Agrawal et al. [174] found a high loading (0.89) for alcohol craving and convergence resulted in an association of SNPs in DRD3 and craving.

While there are many more examples showing promising convergence between GWAS and candidate gene analyses, albeit others showing no convergence, Li et al. [175] performed a meta-analysis of addiction candidate gene association studies and GWAS to investigate functional mechanisms linked to addiction risk. When they compared the lists of genes identified by molecular biological studies of drug-related genes and those by association studies, they observed significantly higher participation in the same gene interaction networks than expected by chance. This work is underscored by Li’s earlier work, the KARG analysis that evaluated 1,500 human genes regulating addictive behaviors and found that these genes significantly impact glutaminergic and dopaminergic pathways [176].

GWAS studies in psychiatry frequently fail to explain a large proportion of variance and non-replication of individual SNPs. Derringer et al. [177] utilizing a “selective scoring” whereby variants (273 SNPs) from eight dopamine-related genes for association with cocaine dependence were considered. They identified a four SNP score significantly associated with the variance. They suggested that (1) limiting SNPs to genes of theoretical importance improves the chances of identifying replicable effects (2) utilizing this scoring approach which considers top-associated SNPs in the aggregate can reveal replicable effects that are too small to be identified at the level of individual SNPs. Along with these precautions we also propose the utilization of “super controls” to eliminate cross contamination of having a control phenotype that also carries the associated disorder especially when considering the complex nature of RDS. Also in developing GARS we are cognizant that the proposed test only provides risk stratification and not actual diagnosis of any disorder including RDS. Most importantly, full comprehension of “psychiatric pharmacogenomics” will undoubtedly involve epigenetic factors, such as DNA histone modifications, for example, methylation and or acetylation that can affect responses to drugs and polymorphic antecedents for either vulnerability or resilience to reward behaviors like RDS, as well as, GWAS convergence to candidate genes [178–185].

Moreover, the utilization of GARS in the clinic has benefits other than just risk stratification, such as, potential therapeutic targets, reduced denial, medical monitoring, for example, in terms of pharmacogenomic tagging [186]. Moreover, we are cognizant that certain ethnic groups will have different rates of polymorphic genes as well different rates of gene polymorphic frequencies [187], and we are developing an algorithm to address this issue involving weighting. In addition, we are analyzing additional data including a total of 393 subjects derived from 9 treatment centers of which 320 have taken the Addiction Severity Index (ASI).

The problem of GWAS is that a general conclusion from GWAS is that most polymorphisms confer little risk increments and explain a small portion of heritability. One example is that 40 loci have been associated with human height, with a known heritability of about 80 %, but those total variants explain only 5 % of the phenotypic variance. It has been suggested that a main problem with GWAS is the need for a level of multiple statistical comparisons that are unprecedented in biology, with stringent analyses to achieve genome-wide significance [186]. In fact, Belcher et al. [188] suggest there is clear evidence to support a genetic basis for SUD. While these authors consider personality as an appropriate endophenotype, we argue that the real endophenotype is RDS as we suggested in the generational study [18]. Certainly, there are a number of studies showing enhanced relapse with specific polymorphisms such as carriers of the DRD2 A1 allele as only one example [189]. Finally, as we pointed out herein, there are fairly recent studies showing GWAS convergence to candidate genes suggesting the continued importance of the candidate approach to identify genetic risk [166–177].

## Conclusion

We encourage further work in the area of psychiatric genetics like GARS and new technology like CARD to track patients during recovery more intensively. While GARS may not be perfect as yet and more research is necessary in the near future, we should not at this point “throw out the baby with the bath water.” Utilization of these suggested new technologies should impact the “revolving door” and secure significant reduction in relapse.
